# Correction of a Disease Mutation using CRISPR/Cas9-assisted Genome Editing in Japanese Black Cattle

**DOI:** 10.1038/s41598-017-17968-w

**Published:** 2017-12-19

**Authors:** Mitsumi Ikeda, Shuichi Matsuyama, Satoshi Akagi, Katsuhiro Ohkoshi, Sho Nakamura, Shiori Minabe, Koji Kimura, Misa Hosoe

**Affiliations:** 1 Institute of Agrobiological Sciences, NARO, Ikenodai 2, Tsukuba, Ibaraki 305-8602 Japan; 2 Institute of Livestock and Grassland Science, NARO, Senbonmatsu 768, Nasushiobara, Tochigi 329-2793 Japan; 3 Institute of Livestock and Grassland Science, NARO, Ikenodai 2, Tsukuba, Ibaraki 305-0901 Japan; 4 Okayama University Graduate School of Environmental and Life Science, Tsushima-Naka 1-1-1, Kita-ku, Okayama 700-8530 Japan

## Abstract

Isoleucyl-tRNA synthetase (IARS) syndrome is a recessive disease of Japanese Black cattle caused by a single nucleotide substitution. To repair the mutated *IARS* gene, we designed clustered regularly interspaced short palindromic repeats (CRISPR)/CRISPR-associated protein 9 (Cas9) to create a double-strand break near the mutation site. CRISPR/Cas9 and donor DNA that contained a synonymous codon for the correct amino acid and an *Aequorea coerulescens* Green Fluorescent Protein (AcGFP) cassette with a piggyBac transposase recognition site at both ends were introduced into bovine fetal fibroblast (BFF) cells isolated from a homozygous mutant calf. Recombinant cells were enriched on the basis of expression of AcGFP, and two cell lines that contained the repaired allele were subcloned. We generated somatic cell nuclear transfer (SCNT) embryos from the repaired cells and transferred 22 blastocysts to recipient cows. In total, five viable fetuses were retrieved at Days 34 and 36. PiggyBac transposase mRNA was introduced into BFF cells isolated from cloned foetuses and AcGFP-negative cells were used for second round of cloning. We transferred nine SCNT embryos to recipient cows and retrieved two fetuses at Day 34. Fetal genomic DNA analysis showed correct repair of the IARS mutation without any additional DNA footprint.

## Introduction

Japanese Black cattle belong to a beef breed that was bred in Japan and is known for high-quality meat distinguished by distinctive marbling. Selective breeding for more than 60 years has yielded high meat quality, but has also resulted in the accumulation of recessive mutations that cause genetic diseases. Given that more than 95% of Japanese Black cattle are bred by artificial insemination, the genetic disorders carried by superior bulls tend to spread latently. One of these disorders, isoleucyl-tRNA synthetase (IARS) syndrome was identified by exome sequencing in 2013^1^. The c.235G>C (p.Val79Leu) substitution in the *IARS* gene causes a 38% reduction in the aminoacylation activity of the IARS protein, which in turn impairs protein synthesis. Homozygous mutant calves exhibit neonatal weakness with intrauterine growth retardation; the birth rate of *IARS* homozygotes is lower than expected from a Mendelian ratio suggesting that affected fetuses are more likely to die prenatally than unaffected fetuses^2^.

Recent remarkable advances in designer nuclease systems, such as zinc finger nucleases (ZFNs)^3^, transcription activator-like effector nucleases (TALENs)^4^, and the clustered regularly interspaced short palindromic repeats (CRISPR)/CRISPR-associated protein 9 (Cas9) system^5–7^_,_ have enabled a site-specific double-strand break to be introduced at a desired locus in genomic DNA. Both ZFNs and TALENs consist of chimeric proteins that contain an adaptable DNA-binding domain fused to the nuclease domain of FokI. The CRISPR/Cas9 system consists of a 20-nucleotide short guide RNA (sgRNA), which hybridizes to the target sequence, and the Cas9 nuclease that originated from *Streptococcus pyogenes*. Given the ease of designing the target sequence for CRISPR/Cas9, this system has been widely used for genome editing in a variety of organisms. Using these designer nuclease systems, many genome-edited livestock animals have been generated^8,9^, not only by gene disruption but also by gene targeting through genome editing-assisted homologous recombination.

The objective of the present study was to generate progeny in which the *IARS* gene had been repaired. The mutated nucleotide in bovine fetal fibroblast (BFF) cells derived from a homozygous mutant fetus was substituted with the correct nucleotide by homologous recombination using CRISPR/Cas9-assisted genome editing. Using these treated BFFs, we were able to obtain cloned fetuses by somatic cell nuclear transfer (SCNT) using recombinant cells in which the mutation had been repaired and foreign marker DNA had been deleted.

## Results

### Isolation of BFF cells

From insemination of a carrier cow by a carrier bull, we derived three fetuses that were retrieved on Day 41 of pregnancy (see Methods). One fetus was diagnosed as C/C (homozygous mutant; #820-1, female), the second as G/C (heterozygous mutant; #820-2, female), and the third as G/G (wild-type; #810, male) with respect to *IARS* genotype. There were no obvious differences in morphology, body weight or length among the different genotypes.

### Assessment of CRISPR/Cas9 target specificity to the mutated IARS gene

In order for the CRISPR/Cas9 system to successfully repair the mutation, it is necessary that the system recognizes the mutated sequence but not the wild-type nor repaired sequence. We designed a protospacer adjacent motif (PAM) sequence that was located at nucleotide 234–236 on the bottom strand of the *IARS* gene, which included the mutated nucleotide 235 G (Fig. 1a). CRISPR/Cas9 activity was assessed using a traffic reporter system. The reporter vector, *p2color*, contains a tdsRed gene driven by a CMV promoter that is located upstream of two tandemly arranged enhanced green fluorescent protein (EGFP) genes with different reading frames. The CRISPR/Cas9 target sequence was inserted between tdsRed and the first EGFP gene, and when a frameshift occurred through the non-homologous end joining pathway after the introduction of a double-strand break by CRISPR/Cas9, EGFP was expressed. Expression of EGFP was only detected when pX330_IARS was introduced with a *p2color* reporter vector that contained the target sequence for the mutant *IARS* (13.6%, Fig. 1b,c) compared to the wild-type (4.8%) and repaired sequences (4.4%), which confirmed the target specificity of pX330_IARS.

**Figure 1:**
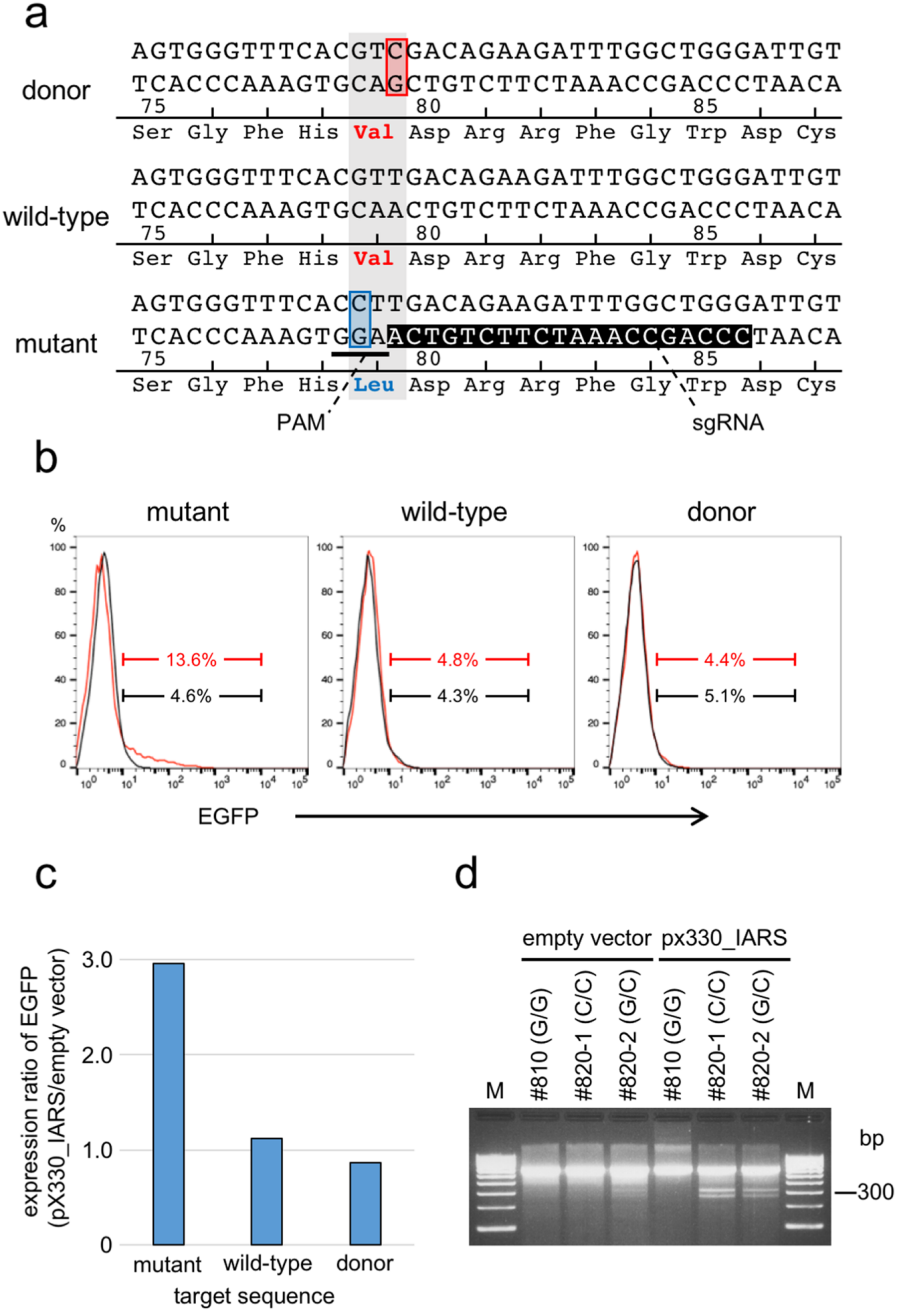
Design of the CRISPR/Cas9 single guide RNA and assessment of target specificity. (**a**) Sequence of the region that flanks the mutation site in IARS. The base at the third position of the codon that encodes p.Val79 was changed in the donor DNA from thymine to cytosine. The PAM sequence (underlined) is located at nucleotides 236 A to 234 G on the bottom strand of the IARS gene and includes the substituted nucleotide 235 G. Inverted characters show the sgRNA. (**b**) Flow cytometric analysis of the target specificity of CRISPR/Cas9. The empty vector pX330 (black line) or pX330_IARS (red line) was introduced into HEK293T cells with the vector *p2color* that contained either the mutant, wild-type or donor target sequence. The histogram shows the expression of EGFP in tdsRed positive cells. **(c)** Comparison of the expression of EGFP for different target sequences. The values are shown as the expression ratios of EGFP when each *p2color* vector was introduced with the empty vector or pX330_IARS. **(d)** Cleavage frequencies in the T7E1 assay in BFF cells derived from #810 (G/G wild-type), #820-1 (C/C homozygous mutant), and #820-2 (G/C heterozygous mutant). M indicates DNA size marker (100 bp ladder).

Next, we performed a T7 endonuclease I (T7E1) mutation mismatch assay^10^ to assess the cleavage specificity and efficiency of pX330_IARS in BFF cells. When the empty vector was introduced, cleaved bands were weakly detected only in BFF cells that were G/C heterozygous (#820-2). When pX330_IARS was introduced, two cleaved bands were observed in BFF cells that were G/C heterozygous (#820-2) or C/C homozygous (#820-1); no cleavage band was detected in wild-type BFF cells (#810) (Fig. 1d). The frequency of cleavage in homozygous mutant BFF cells was slightly higher than in heterozygous mutant BFF cells, suggesting that the designed CRISPR/Cas9 selectively cleaved the target sequence of the mutated *IARS* gene and that an indel mutation occurred in the flanking target region.

### Isolation of recombinant BFF cells

We used the BFF cells from #820-1 and #820-2 for the attempt to repair the mutated *IARS* gene. A CRISPR/Cas9-expressing vector and the donor DNA fragment for *IARS* gene repair were introduced into the BFF cells and, 4–5 days after transfection, recombinant cells were enriched by cell sorting on the basis of *Aequorea coerulescens* Green Fluorescent Protein (AcGFP) expression (Fig. 2b). Five experiments were performed and, in total, 3.5 × 10^6^ cells from #820-1 and 1.9 × 10^7^ cells from #820-2 were transfected, respectively (Table 1). AcGFP-positive colonies were subcloned from each dish and analyzed by junction polymerase chain reaction (PCR; Fig. S1). Two independent recombinant cell lines were isolated from #820-1 and two from #820-2. However, the cell lines from #820-2 both had an ectopic integration and mutation at the *IARS* allele (Table 1). One of the recombinant cell lines from #820-1, termed #820-1-2, showed sufficient proliferation and precise recombination by Southern blot analysis (Fig. 2c). Thus, we used this cell line as the donor cells to generate the first round of clones.

**Figure 2:**
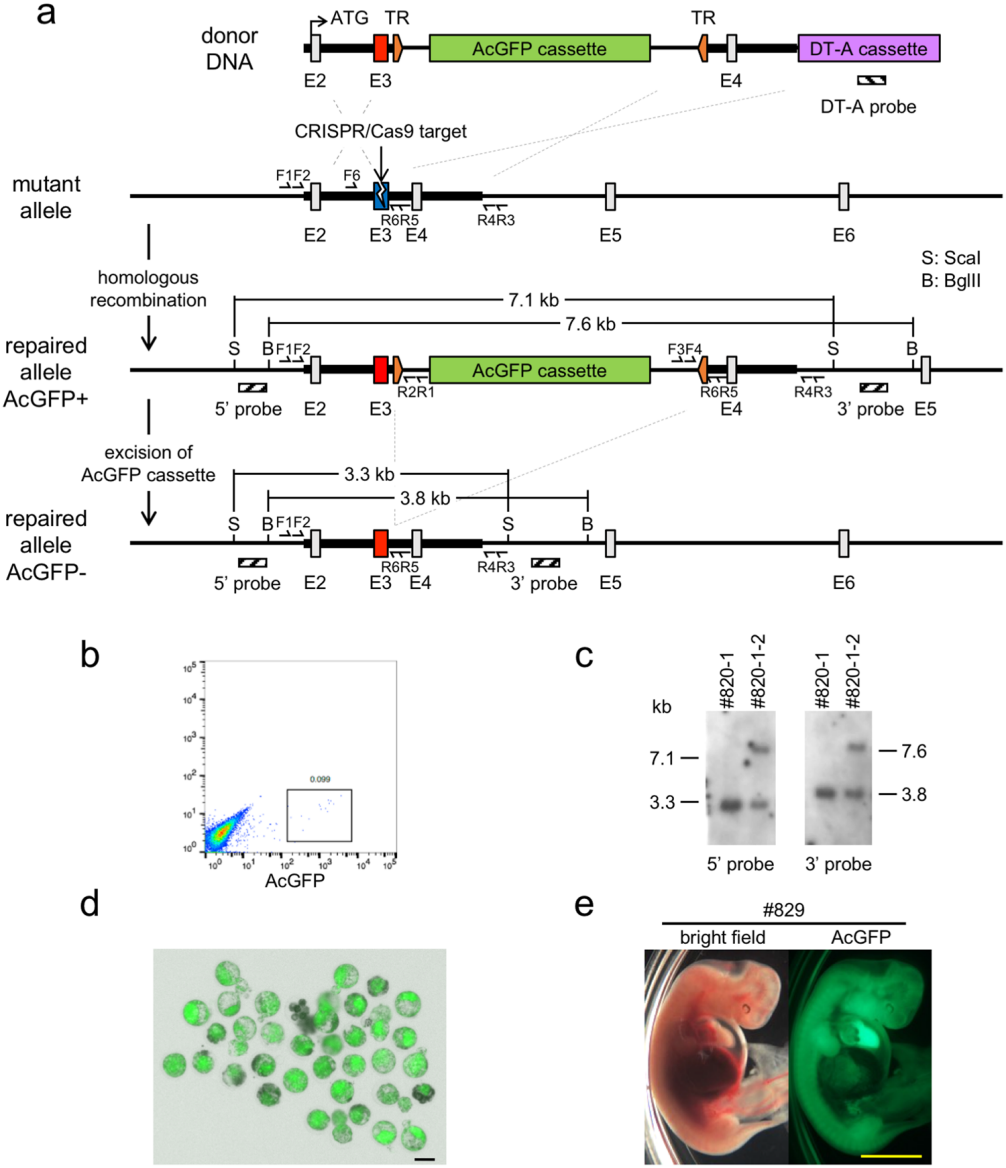
CRISPR/Cas9-assisted homologous recombination at the mutated IARS locus. (**a**) Schematic diagram of the strategy for CRISPR/Cas9-assisted homologous recombination. The mutation site is located in exon 3 (E3). The striped boxes show the positions of the probes for Southern blot analysis. TR indicates recognition sites for the piggyBac transposase. F1-4 and R1-6 refer to the primer sites for junction and substitution analysis, respectively. F5 and R6 are used for T7E1 assay (see Supplemental Table 1). (**b**) Enrichment of AcGFP-positive cells by cell sorting. (**c**) Southern blot analysis of subcloned recombinant cell lines. #820-1 is the parent cell line of #820-1-2. (**d**) Day 6 SCNT embryos derived from #820-1-2. The image shown is a bright-field image merged with a fluorescent image. Bar = 100 µm. (**e**) Bright-field and fluorescent images of a Day 34 cloned fetus. Bar = 5 mm.

**Table 1:** Subcloning of recombinant cells for clone generation.

Experimental No.	Parental cells (genotype)	No. of cells	No. of recombinant clones	Random integration	Unexpected mutation
1	820-1 (C/C)	1.5 × 10^6^	1	−	−
	820-2 (G/C)	1.5 × 10^6^	0		
2	820-1 (C/C)	2.0 × 10^6^	1	−	−
	820-2 (G/C)	2.0 × 10^6^	1	−	+
3	820-2 (G/C)	3.0 × 10^6^	1	+	+
4	820-2 (G/C)	6.0 × 10^6^	0		
5	820-2 (G/C)	6.0 × 10^6^	0		+

### Generation of the first round of cloned fetuses derived from recombinant BFF cells

A rate of fusion of #820-1-2 cells with enucleated oocytes of 91.2% was obtained and 68.0% of embryos developed to the blastocyst stage (Table 2). The SCNT embryos exhibited strong expression of AcGFP from the reporter cassette (Fig. 2d) and 22 embryos that showed normal development were transferred into recipient cows; each recipient cow receive two embryos. Ultrasound diagnosis at Day 32 detected the presence of fetuses in six of the 11 recipient cows (#780, #782, #829, #832, #869, and #931); we retrieved two groups of four fetuses from three recipient cows at Day 34 (#782-1, -2, #832, and #869) and Day 36 (#780-1, -2, #829, and #931), respectively. Unfortunately, #782-1, -2 and #832 seemed to have died just before recovery; therefore, we obtained five viable fetuses. These fetuses appeared to be morphologically normal and showed strong expression of AcGFP, as at the blastocyst stage (Fig. 2e, S2

**Table 2:** *In vitro* development of SCNT embryos.

Round of cloning	Donor cell line	No. of enucleated oocytes (%)	No. of fused oocytes (%)	No. of cultured embryos	No. of cleaved embryos (%)	No. of blastocysts (%)		
						Day 6	Day 7	Total
1st	#820-1-2	57/62 (91.9)	52/57 (91.2)	50	41 (82.0)	23 (46.0)	11 (22.0)	34 (68.0)
2nd	#829	44/54 (81.5)	21/24 (87.5)	20	14 (70.0)	6 (30.0)	1 (5.0)	7 (35.0)
	#869		16/20 (80.0)	15	8 (53.3)	4 (26.7)	1 (6.7)	5 (33.3)
	total		37/44 (84.1)	35	22 (62.9)	10 (28.6)	2 (5.7)	12 (34.3)

).

### Generation of the second round of 2nd cloned fetuses with no foreign DNA

To excise the AcGFP reporter cassette, piggyBac transposase mRNA was introduced into BFF cells isolated from #829 and #869 cloned fetuses (described above). Five days after transfection, 1.9% of transfected cells showed 10–100-fold decrease in the level of AcGFP expression (Fig. 3a) and direct sequence analysis of a PCR fragment from the junction region for these cells confirmed that the AcGFP reporter cassette was excised correctly as designed. We enriched the population of #829 and #869 cells that contained low levels of AcGFP by cell sorting, and used these as donor cells for a second round of SCNT. The rates of fusion of #829 and #869 cells with enucleated oocytes were 87.5% and 80%, respectively, and the proportions of embryos that developed to the blastocyst stage on Day 6 were 30.0% and 26.7%, respectively (Table 2). Six and four embryos derived from #829 and #869 cells, respectively, were transferred into nine recipient cows, and two fetuses (#675 and #687) derived from #829 cells were retrieved at Day 34. These fetuses appeared to be morphologically normal and showed no expression of AcGFP (Figs 3b, S2). Southern blot analysis demonstrated that these fetuses did not carry the DT-A cassette for negative selection (Fig. S3). Furthermore, bands of 7.1 kb (5′ probe) and 7.6 kb (3′ probe) that were detected in #829 cells were not present in BFF cells from cloned fetuses #675 and #687 from the second round of SCNT (Fig. 3c). Direct sequence analysis of the genomic PCR product that corresponded to the mutation site in #829 cells only detected the mutant allele (Fig. 3d, Left), because the AcGFP cassette inhibited amplification from the repaired allele. In contrast, overlapping peaks at c.235 G/C and c.237 T/C (Fig. 3d, Left) were observed and no indel mutations were detected at the junction site (Fig. 3d, Right) in #687 cells, which suggests that the mutation had been repaired and the AcGFP cassette excised as designed. Next, we estimated the amount of mRNA transcription from the repaired allele by direct sequencing of the RT-PCR product. In #829 cells, very little mRNA was transcribed from the repaired allele because of aberrant splicing, which might be caused by the AcGFP cassette (Fig. 3d, Middle). In contrast, in #687 cells, almost equal amounts of transcript were transcribed from the two alleles (Fig. 3d, Middle), which suggests that IARS enzymatic activity was restored to the normal level in these fetuses.

**Figure 3:**
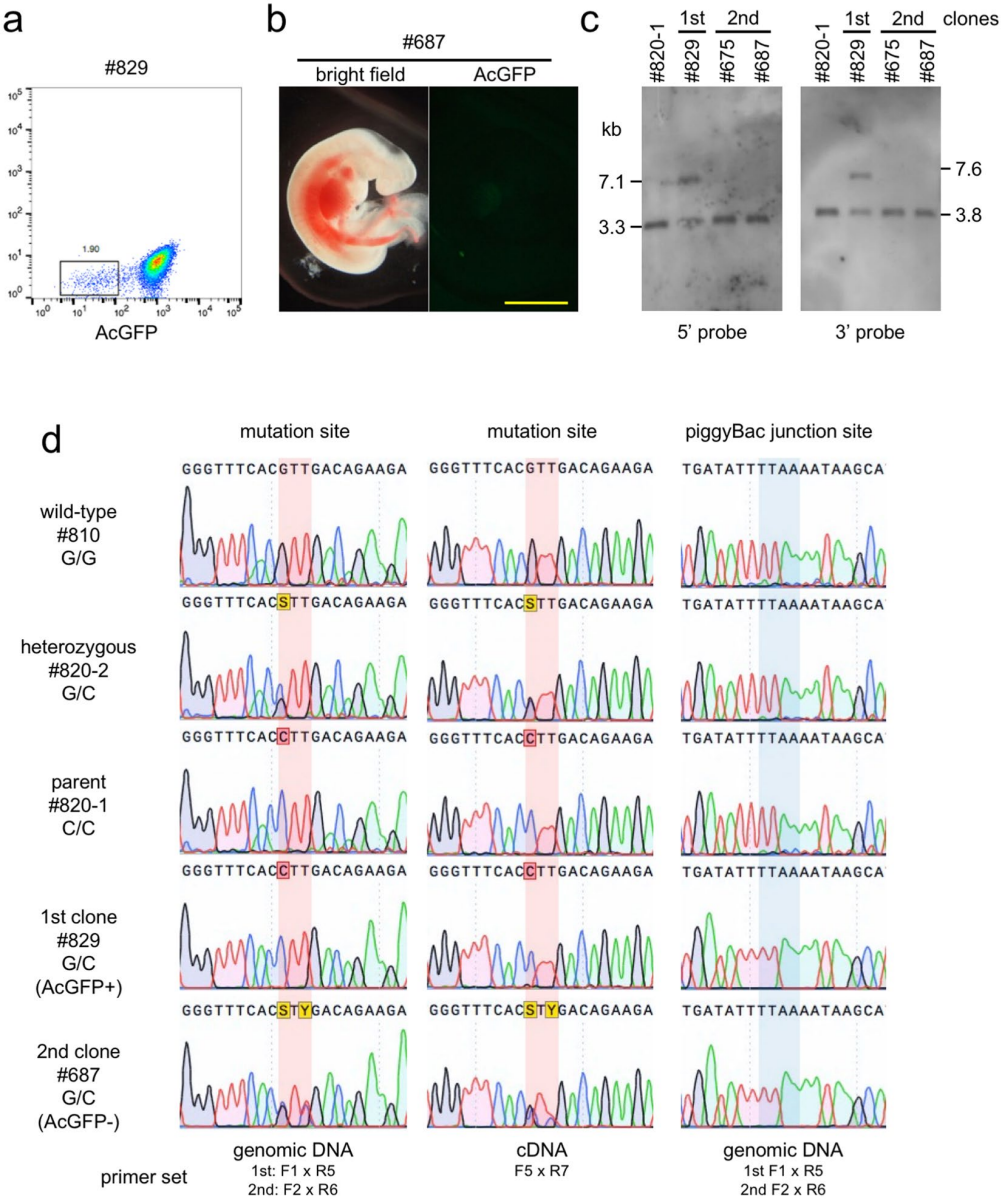
Analysis of cloned fetuses from the second round of SCNT. (**a**) Isolation of a population of cells with low levels of AcGFP expression from #829 cells into which piggyBac transposase mRNA had been introduced. (**b**) Bright-field and fluorescent images of a Day 34 cloned fetus from the second round. Bar = 5 mm. (**c**) Southern blot analysis of cloned fetuses from the first and second round of SCNT. #820-1 is the parental BFF cell. (**d**) Sequence analysis of the region that flanks the repaired IARS locus. Left: Direct sequencing of PCR products that correspond to the mutation site. Amplification from the repaired allele in the #829 cells failed due to the presence of the AcGFP cassette. Middle: Direct sequencing of RT-PCR products. In #829 cells, very little mRNA was transcribed from the repaired allele because of aberrant splicing, which might be caused by the AcGFP cassette. Right: Direct sequencing of PCR products that correspond to the piggyBac junction site.

A search for off-target effects of the sgRNA against bovine genomic DNA revealed that there was no 20 mer perfect match site but identified twenty 12 mer match sites (Table S2). We attempted to analyze the sequences of the 12 mer match sites in the genomic DNA of the parent cells, #820-1, and in fetus #687 from the second round of cloning. We amplified sequences from 17 sites by PCR and no mutations were detected in the region 50 bp upstream and downstream from the predicted target sites (Table S3).

## Discussion

In this study, we succeeded in substituting a single mutated nucleotide in the *IARS* gene with the appropriate nucleotide using CRISPR/Cas9-assisted genome editing. This procedure has the potential to restore genetic diversity and to enable the genetic resources of livestock to be utilized effectively. In particular, it could have innovative applications for genetic improvement for genetic improvement in livestock that have a long gestation cycle, such as cattle. Through the recent remarkable progress in genome editing technology, many genome-edited livestock have been generated^9,11^. For example, given the similarity of pigs to humans in terms of size and physiology, genetically modified pigs have been developed as disease models and organ donors for xenotransplantation. Although there is a lower demand for gene disruption in ruminant livestock than in pigs, hornless dairy cows^12^ have been generated, as well as cows and bucks that do not produce milk allergen^13–15^. In addition to gene targeting, genome editing-mediated homologous recombination has been used to generate goats that produce human lactoferrin^16^ and cows that produce human serum albumin^17^ in milk instead of inherent milk proteins. Liu *et al*. succeeded in developing cows that produced the antiviral proteins lysostaphin^18^ and human lysozyme^19^ in the mammary gland by zinc-finger nuclease-assisted gene targeting, and the milk produced by these transgenic cows showed antiviral activity *in vitro*. These strategies show the feasibility of genome editing technologies in reducing the costs and requirement for animal healthcare in animal husbandry and also in creating new biological industries.

Although we succeeded in repairing the mutated gene using genome editing, there is still room for improvement in the approach used. Firstly, we produced progeny using SCNT technology. SCNT is an innovative technique for reproducing animals from non-gamete cells, but there are issues with low efficiency and high developmental mortality caused by deficient epigenetic reprograming. In addition, passage number of the donor cells needs to be minimal to avoid abnormalities in the cloned embryos^20–25^, but this limitation makes it more difficult to select donor cells that have undergone the correct repair. Initially, we intended to remove the AcGFP cassette before the first round of SCNT; however, we abandoned this plan because of growth retardation of subcloned cells following the enrichment by cell sorting. The most widely used method for genome editing in mice is embryo microinjection, but a higher efficiency system is needed to accomplish homologous recombination in livestock, especially monotocous species such as cattle. Another possible route to generate progeny is to utilize pluripotent cells such as embryonic stem cells or induced pluripotent stem cells. Hayashi *et al*. reported that primordial germ cell-like cells that had been induced from mouse pluripotent cells *in vitro* could differentiate into gametes and could be used to produce progeny^26–28^. Attempts are being made to establish pluripotent stem cells from livestock^29–32^, and reconstitution of the germ line from genome-modified pluripotent stem cells *in vitro* would dramatically shorten the cycle for the improvement of livestock.

The second problem is the introduction of unexpected modifications in the genomic DNA. The strategy used here was designed so that the second round of cloned fetuses that carried the repaired *IARS* gene would not contain any foreign DNA; however, we used the AcGFP reporter cassette to aid the initial selection of recombinant cells. PCR and Southern blot analysis did not detect the foreign DNA in the genomic DNA of the second round of cloned fetuses, but it could not completely exclude the introduction of the small, fragmented foreign DNA. In addition, off-target effects in designer nuclease systems have to be addressed. We confirmed the target specificity of CRISPR/Cas9, but the possibility that the nuclease also induced off-target mutagenesis could not be ruled out. Recently, Gao *et al*. reported a novel gene insertion system with reduced off-target effects using single Cas9 nickase and chromatin immunoprecipitation (ChIP) ChiP-seq analysis^33^. To ensure the safety of the approach and public acceptance, further improvement of nuclease specificity and efficient detection of off-target mutagenesis are required for practical application in livestock breeding^34^.

In conclusion, our results show that the application of genome editing to livestock improvement could prevent the wastage of excellent genome resources. It also could provide a more rapid innovative method for livestock breeding.

## Methods

### Animal care

All procedures involving the care and use of animals were approved by the Animal Research Committee of the National Agriculture and Food Research Organization. Cows were treated in accordance with the Fundamental Guidelines for Proper Conduct of Animal Experiment and Related Activities in Academic Research Institutions under the jurisdiction of the Ministry of Education, Culture, Sports, Science and Technology of Japan.

### Isolation of BFF cells carrying the IARS mutation

A carrier Japanese Black cow was artificially inseminated with semen from a carrier bull, and Day 7 (Day 0 = the day of estrus) embryos at the morula or blastocyst stages were collected by nonsurgical uterine flushing^35^. The recovered embryos were frozen by conventional methods^36^ and stored in liquid nitrogen. Thawed embryos were transferred to a uterine horn ipsilateral to the corpus luteum (CL) of estrus-synchronized recipient cows. A total of six embryos were transferred and pregnancy was diagnosed by ultrasonography. On Day 41, two pregnant cows were sacrificed by administering an overdose of sodium pentobarbital and three fetuses were recovered. Fibroblast cells derived from the recovered fetuses were genotyped by PCR and maintained in Dulbecco’s modified Eagle’s medium (DMEM; Nacalai Tesque) supplemented with 10% fetal bovine serum (FBS) under 5% CO_2_ and 95% air at 37 °C.

### Design of a CRISPR/Cas9 single guide RNA and assessment of activity

A CRISPR/Cas9 sgRNA was designed to introduce a double-strand DNA break near the mutation site in the *IARS* gene (Fig. 1a). The PAM was positioned at nucleotides 236 A to 234 G on the bottom strand, which included the substituted nucleotide 235 G. The oligo DNA for the sgRNA was cloned into the BbsI site of the pX330 vector (Addgene) and the cleavage activity was estimated using a traffic reporter system that employed the *p2color* vector^37^. Briefly, 1 µg of pX330 plasmid containing the sgRNA sequence (pX330_IARS) was mixed with 1 µg of *p2color* vector that contained either the mutant, wild-type or repaired target sequence. HEK293T cells were placed in a 10-mm gap cuvette with the mixed DNA samples and subjected to electroporation using a NEPA21 Electroporator (NEPA GENE). The electroporation conditions were as follows: 1) poring pulse: input voltage, 150 V; pulse width/interval, 5/50 ms; pulse number, 2; 2) transfer pulse: input voltage, 20 V; pulse width/interval, 50/50 ms; pulse number, 5. After two days, expression of EGFP in the cells was analyzed using a MoFlo Astrios cell sorter (Bechman Coulter). For the T7E1 assay, 5 µg of pX330 (empty vector) or pX330_IARS plasmid were introduced into BFF cells with 1 µg of CAG promoter-driven AcGFP expression vector using a NEPA21 Electroporator (the electroporation conditions were the same as above). Two days after electroporation, AcGFP-positive cells were sorted using a MoFlo Astrios cell sorter, and genomic DNA was extracted using a NucleoSpin Tissue kit (Macherey-Nagel). The T7E1 assay was performed using a T7 endonuclease I assay kit (GeneCopoeia) in accordance with the manufacturer’s protocol. The primers for PCR are listed in Table S2.

### Off-target analysis

The search for off-target effects by the sgRNA sequence was performed using a web server for selecting rational CRISPR/Cas targets (CRISPRdirect^38^, https://crispr.dbcls.jp) against bovine genome database [Cow genome, Bos_taurus_UMD_3.1.1/bosTau8 (Jun, 2014)]. The genomic region flanking the predicted off-target sites were amplified by PCR and sequences were determined by direct sequencing with each forward or reverse PCR primer. The primers for PCR are listed in Table S3.

### Design of donor DNA

The knock-in vector consisted of two arms that were homologous to the 1.0 kb of genomic DNA upstream and downstream of the mutation site in the *IARS* gene, respectively, a reporter cassette for AcGFP (Takara Bio) with the piggyBac transposase recognition site (TR) at both ends, and a Diphtheria toxin A fragment (DT-A) cassette for negative selection (Fig. 2a). the AcGFP cassette was inserted into the third intron of the TTAA piggyBac transposase target site. To distinguish the repaired IARS locus from the mutant and wild-type loci, the base at the third position of the codon that encoded p.Val79 was changed in the donor DNA from thymine to cytosine (Fig. 1a).

### Repair of the mutant IARS gene in BFF cells

BFF cells that were derived from an IARS homozygous or heterozygous mutant and had undergone less than five passages were trypsinized and 5 × 10^5^ cells were suspended in 25 µl electroporation buffer (137 mM NaCl, 5 mM KCl, 0.7 mM Na_2_HPO_4_, 6 mM glucose, 25 mM HEPES) with 0.5 µg of pX330_IARS and 0.2 µg of donor DNA fragment. The cells were then placed in a 4-mm gap cuvette and subjected to electroporation using a BTX ECM600 Electro Cell Manipulator (BTX Harvard Apparatus). The electroporation conditions were as follows: input voltage: 150 V; capacitance: 0.5 µF; pulse number, 1. After electroporation, the cells were diluted immediately with culture medium and each sample was plated onto an individual 35-mm dish. Six to seven days after electroporation, AcGFP-positive cells were enriched using a MoFlo Astrios cell sorter and approximately 500 cells were plated onto a 90-mm dish. After two weeks, growing colonies were subcloned and homologous recombination was confirmed by junction PCR and Southern blot analysis. The primers for PCR are listed in Table S1.

### Production of SCNT embryos

SCNT was performed as described previously^22,39^. Briefly, following *in vitro* maturation culture, the cumulus cells surrounding the oocytes were removed by vortex mixing in 0.1% hyaluronidase (Sigma-Aldrich), and oocytes with a first polar body that was visible under a dissection microscope were enucleated. An IARS-repaired BFF cell was transferred into the perivitelline space of an enucleated oocyte. The oocyte–cell complex was sandwiched between a pair of electrodes in Zimmerman mammalian cell fusion medium^40^ and a single direct current pulse of 25 V was applied for 10 µs for oocyte-cell fusion. The SCNT embryos were chemically activated 1 h after fusion by treatment with 10 µM A23187 (a calcium ionophore; Calbiochem, Merck KGaA) in PBS for 5 min, followed by 2.5 µg/ml cytochalasin D (Sigma-Aldrich) and 10 µg/ml cycloheximide (Sigma-Aldrich) in TCM-199 with 10% FBS for 1 h, and then 10 µg/ml cycloheximide alone for 4 h. After chemical activation, the SCNT embryos were cultured in CR1aa medium^41^ that contained 3 mg/mL bovine serum albumin (fatty acid free; Sigma-Aldrich) for 48 h under 5% CO_2_, 5% O_2_, and 90% N_2_ at 38.5 °C. Cleaved embryos were then transferred into CR1aa medium supplemented with 5% FBS and cultured until Day 6 or 7 (Day 0 = the day of SCNT).

### Embryo transfer and recovery of cloned fetuses

Day 6 SCNT embryos with normal morphology were transferred to a uterine horn ipsilateral to the corpus luteum of estrus-synchronized recipient cows. Pregnancy was diagnosed by ultrasonography, and pregnant cows were sacrificed on Day 34 or 36 by administering an overdose of sodium pentobarbital. Fibroblast cells isolated from the retrieved fetuses were maintained in Dulbecco’s modified Eagle’s medium (DMEM; Nacalai Tesque) supplemented with 10% FBS under 5% CO_2_ and 95% air at 37 °C.

### Excision of the AcGFP cassette using the piggyBac system

The BFF cells derived from cloned fetuses from the first round of SCNT carried the AcGFP cassette for use in positive selection; to remove this cassette, piggyBac transposase mRNA was introduced. BFF cells that had undergone less than three passages were trypsinized and suspended (1 × 10^6^ cells) in 100 µl of Opti-MEM (Thermo Fisher Scientific) with 5 µg of Excision-only piggyBac transposase mRNA (System Biosciences). Cells were placed in a 10-mm gap cuvette and subjected to electroporation using a NEPA21 electroporator (NEPA GENE). The electroporation conditions were as follows: 1) poring pulse: input voltage, 150 V; pulse width/interval, 5/50 ms; pulse number, 2; 2) transfer pulse: input voltage, 20 V; pulse width/interval, 50/50 ms; pulse number, 5. After electroporation, cells were diluted immediately with culture medium and plated onto 60-mm dishes. Five days after electroporation, cells with low AcGFP expression were enriched using a MoFlo Astrios cell sorter and, after several passages, cells that were negative for AcGFP were used for SCNT to generate a second round of clones.

### PCR, sequencing, and Southern blot analysis

Genomic DNA was extracted from BFF cells using a NucleoSpin Tissue kit and PCR amplification was performed using PrimeStar HS DNA Polymerase (Takara Bio). For reverse transcription (RT)-PCR analysis, total RNA was prepared from cells using an RNeasy Micro kit (QIAGEN) with RNase-free DNase treatment. First-strand cDNA was synthesized from 1.0 µg of total RNA using a 1st strand synthesis kit (Takara Bio) and aliquots of 1/10-1/50 of the cDNA products were used for RT-PCR analysis. The PCR primers that were used for junction PCR and RT-PCR analysis are shown in Fig. 2a and Table S1. The PCR products were directly sequenced using the BigDye Terminator v3.1 Cycle Sequencing Kit and ABI 310 Genetic Analyzer (Thermo Fisher Scientific). Homologous recombination was assessed by Southern blot analysis. Two µg of genomic DNA that had been digested with ScaI or BglII were separated on 0.8% agarose gels and transferred onto nylon membranes. Digoxigenin (DIG)-labelled DNA probes (Fig. 1a) were made by using PCR DIG Labelling Mix (Roche) with the primers listed in Table S1. Hybridization and DIG detection were performed using the DIG detection system (Roche) in accordance with the manufacturer’s protocol. The CDP-Star Detection Reagent (Roche) was used to develop the membrane and the resulting chemiluminescent signal was detected using Amersham Hyperfilm ECL (GE Healthcare).

## Electronic supplementary material

Supplementary information
