# Publisher Correction: Correction of a Disease Mutation using CRISPR/Cas9-assisted Genome Editing in Japanese Black Cattle

**DOI:** 10.1038/s41598-018-19725-z

**Published:** 2018-01-19

**Authors:** Mitsumi Ikeda, Shuichi Matsuyama, Satoshi Akagi, Katsuhiro Ohkoshi, Sho Nakamura, Shiori Minabe, Koji Kimura, Misa Hosoe

**Affiliations:** 1 Institute of Agrobiological Sciences, NARO, Ikenodai 2, Tsukuba, Ibaraki 305-8602 Japan; 2 Institute of Livestock and Grassland Science, NARO, Senbonmatsu 768, Nasushiobara, Tochigi 329-2793 Japan; 3 Institute of Livestock and Grassland Science, NARO, Ikenodai 2, Tsukuba, Ibaraki 305-0901 Japan; 4 Okayama University Graduate School of Environmental and Life Science, Tsushima-Naka 1-1-1, Kita-ku, Okayama 700-8530 Japan

Correction to: *Scientific Reports* 10.1038/s41598-017-17968-w, published online 19 December 2017

The original version of this Article contained an error in Table 2 where the following text was inadvertently inserted:

“*Please centre-align all of the text in this table.*”

This error has now been corrected in the HTML and PDF versions of the Article.

