# Supplementary material for: Correction of a Disease Mutation using CRISPR/Cas9-assisted Genome Editing in Japanese Black Cattle
Source: Sci Rep. 2017 Dec 19;7:17827. doi: 10.1038/s41598-017-17968-w (PMC5736618; doi:10.1038/s41598-017-17968-w)
Supplement: Supplementary file 1 — Supplementary information [file 41598_2017_17968_MOESM1_ESM.pptx]

## Slide 1
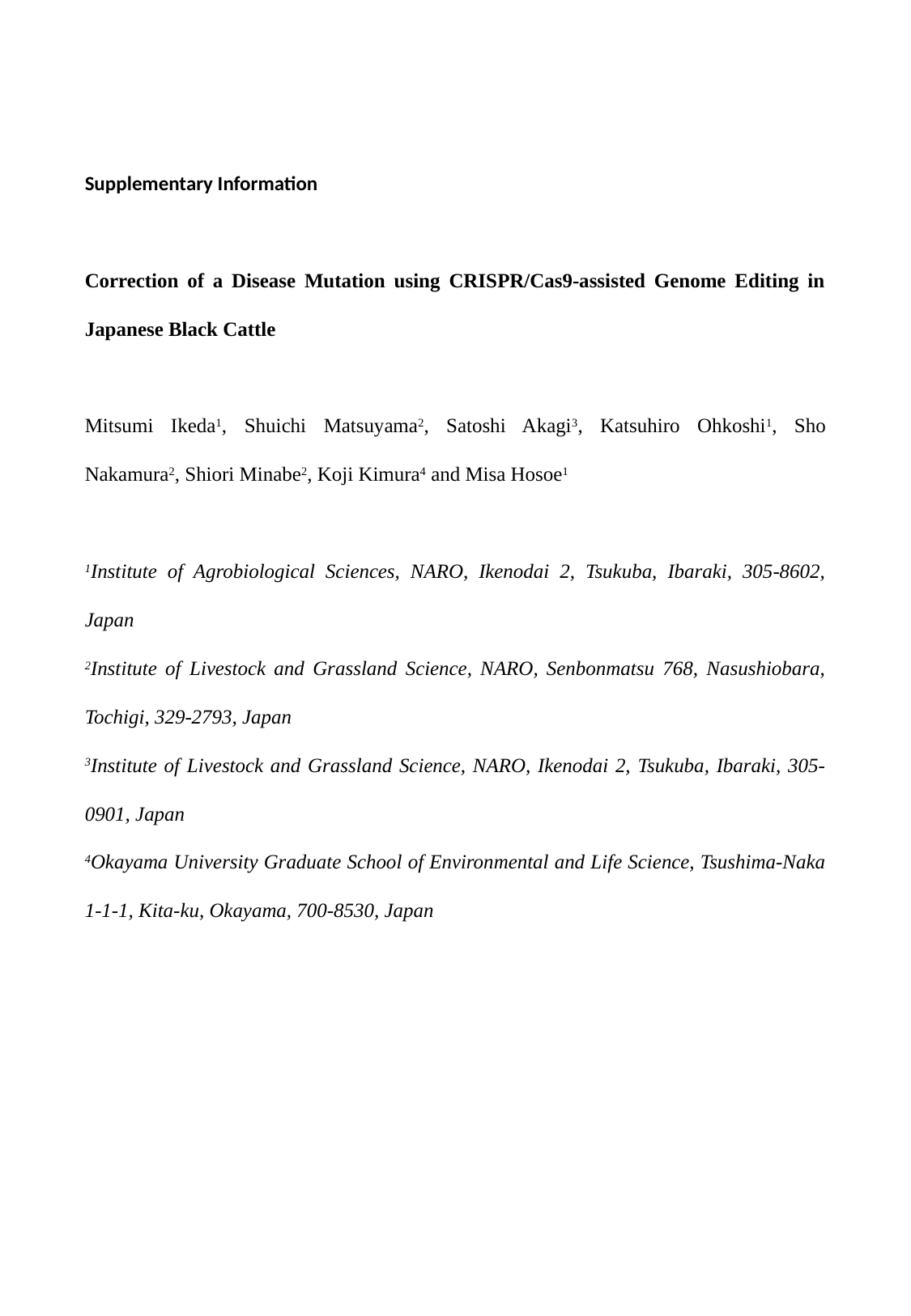

Supplementary Information
Correction of a Disease Mutation using CRISPR/Cas9-assisted Genome Editing in Japanese Black Cattle
Mitsumi Ikeda1, Shuichi Matsuyama2, Satoshi Akagi3, Katsuhiro Ohkoshi1, Sho Nakamura2, Shiori Minabe2, Koji Kimura4 and Misa Hosoe1
1Institute of Agrobiological Sciences, NARO, Ikenodai 2, Tsukuba, Ibaraki, 305-8602, Japan
2Institute of Livestock and Grassland Science, NARO, Senbonmatsu 768, Nasushiobara, Tochigi, 329-2793, Japan
3Institute of Livestock and Grassland Science, NARO, Ikenodai 2, Tsukuba, Ibaraki, 305-0901, Japan
4Okayama University Graduate School of Environmental and Life Science, Tsushima-Naka 1-1-1, Kita-ku, Okayama, 700-8530, Japan

## Slide 2
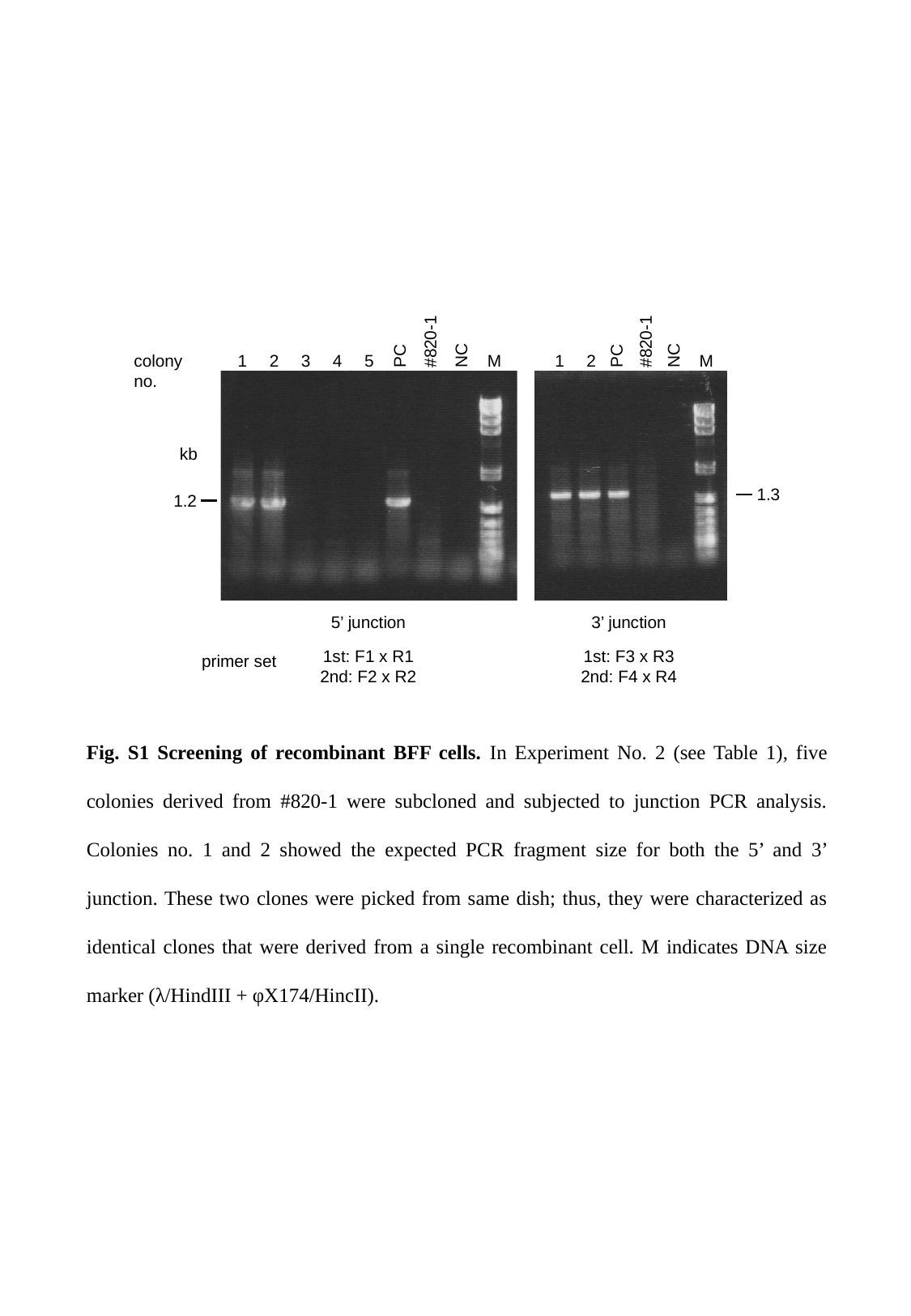

#820-1
#820-1
NC
NC
PC
PC
colony no.
1
2
3
4
5
M
1
2
M
kb
1.3
1.2
5’ junction
3’ junction
1st: F1 x R1
2nd: F2 x R2
1st: F3 x R3
2nd: F4 x R4
primer set
Fig. S1 Screening of recombinant BFF cells. In Experiment No. 2 (see Table 1), five colonies derived from #820-1 were subcloned and subjected to junction PCR analysis. Colonies no. 1 and 2 showed the expected PCR fragment size for both the 5’ and 3’ junction. These two clones were picked from same dish; thus, they were characterized as identical clones that were derived from a single recombinant cell. M indicates DNA size marker (λ/HindIII + φX174/HincII).

## Slide 3
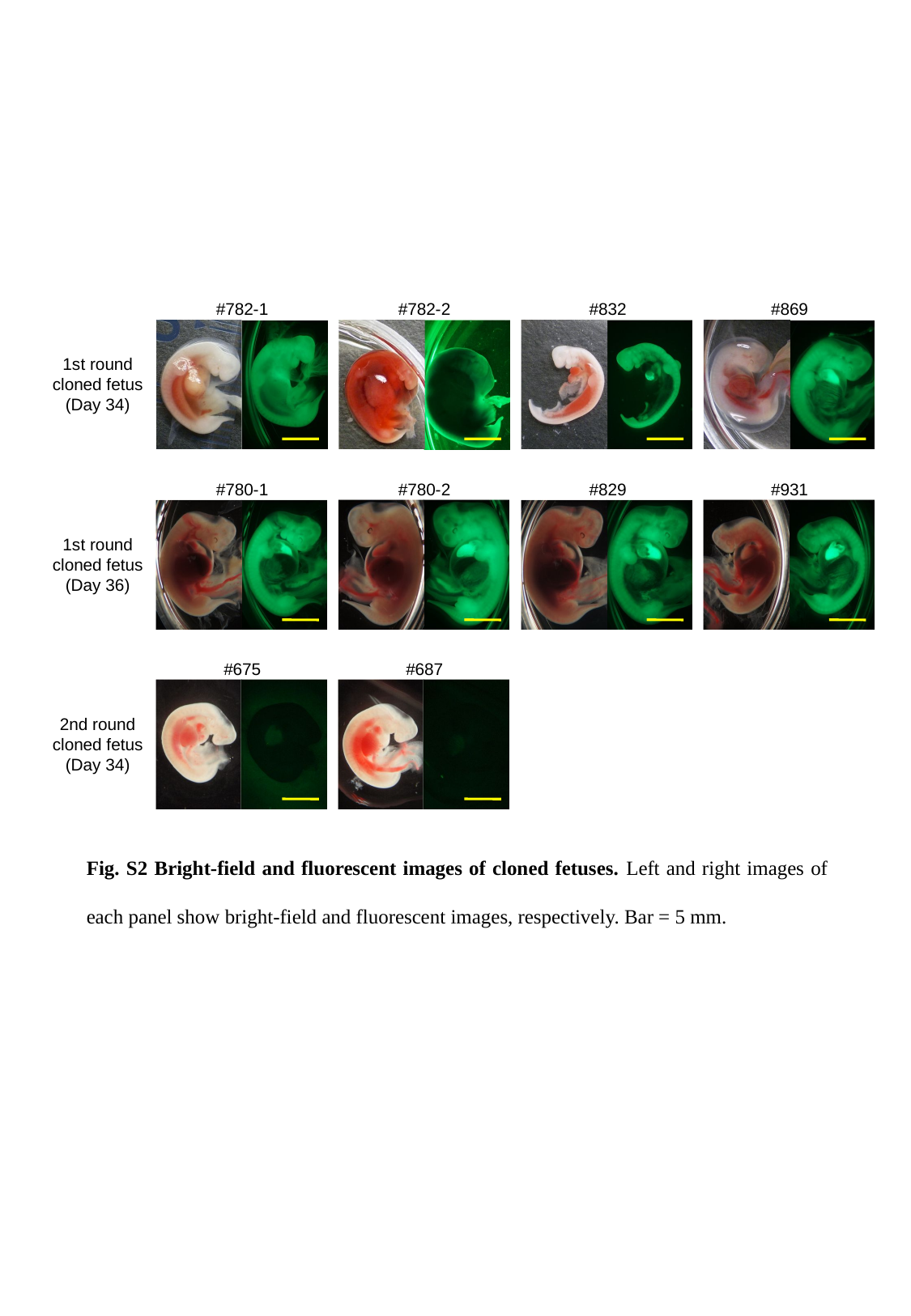

d34
#782-1
#782-2
#832
#869
1st round
cloned fetus
(Day 34)
#780-1
#780-2
#829
#931
1st round
cloned fetus
(Day 36)
#675
#687
2nd round
cloned fetus
(Day 34)
Fig. S2 Bright-field and fluorescent images of cloned fetuses. Left and right images of each panel show bright-field and fluorescent images, respectively. Bar = 5 mm.

## Slide 4
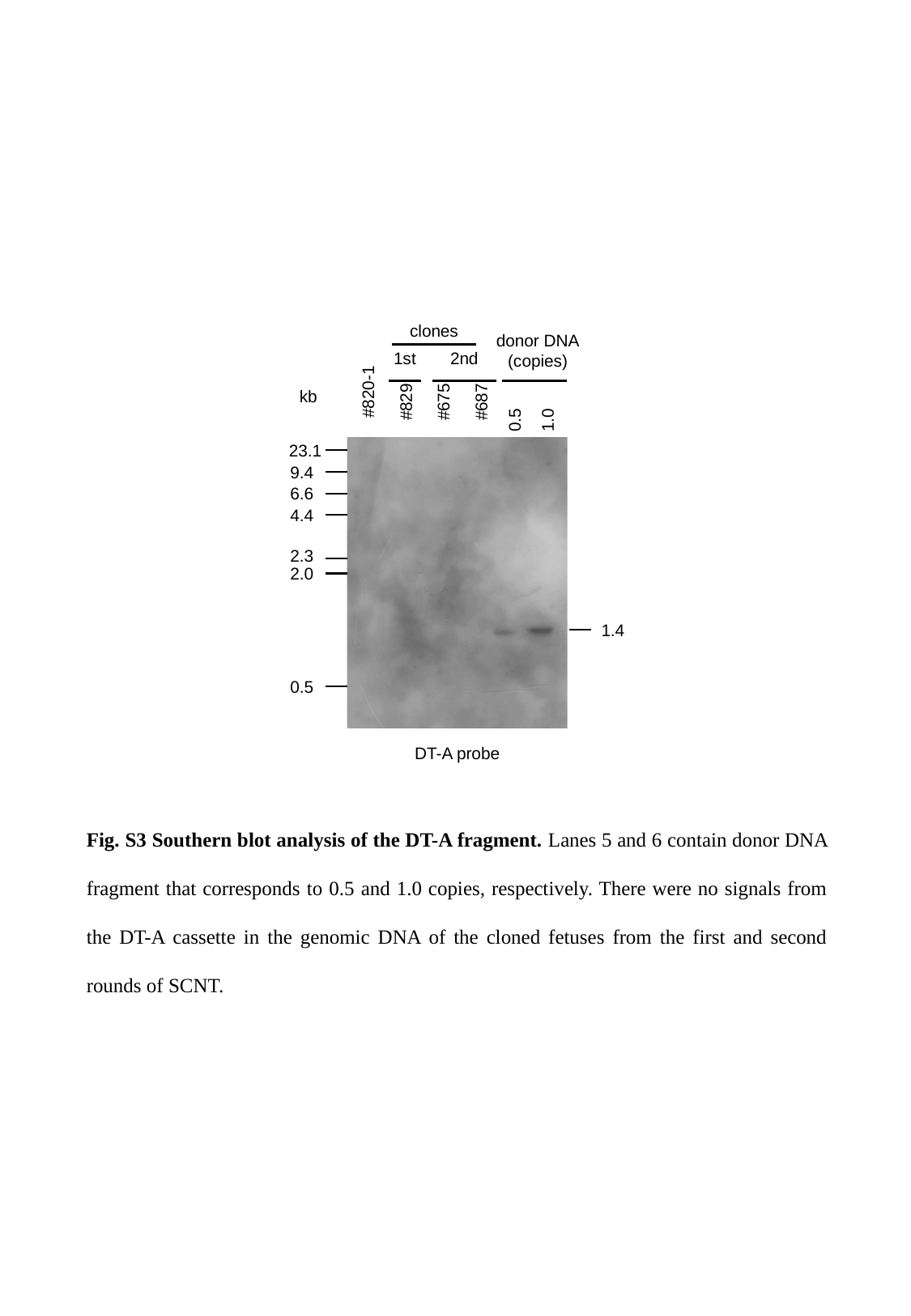

clones
donor DNA
(copies)
1st
2nd
#820-1
kb
#829
#675
#687
0.5
1.0
23.1
9.4
6.6
4.4
2.3
2.0
0.5
1.4
DT-A probe
Fig. S3 Southern blot analysis of the DT-A fragment. Lanes 5 and 6 contain donor DNA fragment that corresponds to 0.5 and 1.0 copies, respectively. There were no signals from the DT-A cassette in the genomic DNA of the cloned fetuses from the first and second rounds of SCNT.

## Slide 5
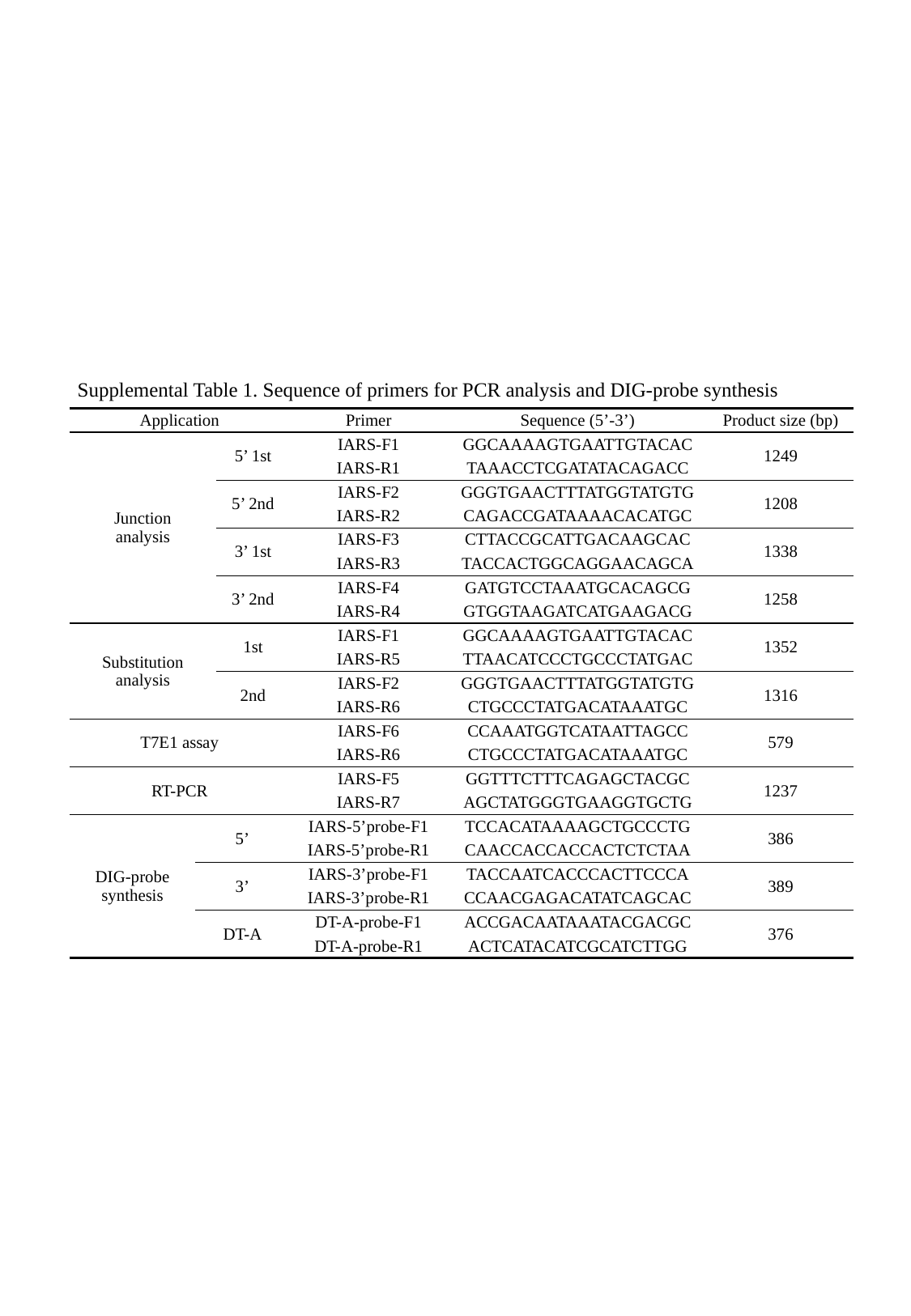

| Supplemental Table 1. Sequence of primers for PCR analysis and DIG-probe synthesis | | | | | |
| --- | --- | --- | --- | --- | --- |
| Application | | | Primer | Sequence (5’-3’) | Product size (bp) |
| Junction analysis | | 5’ 1st | IARS-F1 | GGCAAAAGTGAATTGTACAC | 1249 |
| | | | IARS-R1 | TAAACCTCGATATACAGACC | |
| | | 5’ 2nd | IARS-F2 | GGGTGAACTTTATGGTATGTG | 1208 |
| | | | IARS-R2 | CAGACCGATAAAACACATGC | |
| | | 3’ 1st | IARS-F3 | CTTACCGCATTGACAAGCAC | 1338 |
| | | | IARS-R3 | TACCACTGGCAGGAACAGCA | |
| | | 3’ 2nd | IARS-F4 | GATGTCCTAAATGCACAGCG | 1258 |
| | | | IARS-R4 | GTGGTAAGATCATGAAGACG | |
| Substitution analysis | | 1st | IARS-F1 | GGCAAAAGTGAATTGTACAC | 1352 |
| | | | IARS-R5 | TTAACATCCCTGCCCTATGAC | |
| | | 2nd | IARS-F2 | GGGTGAACTTTATGGTATGTG | 1316 |
| | | | IARS-R6 | CTGCCCTATGACATAAATGC | |
| T7E1 assay | | | IARS-F6 | CCAAATGGTCATAATTAGCC | 579 |
| | | | IARS-R6 | CTGCCCTATGACATAAATGC | |
| RT-PCR | | | IARS-F5 | GGTTTCTTTCAGAGCTACGC | 1237 |
| | | | IARS-R7 | AGCTATGGGTGAAGGTGCTG | |
| DIG-probe synthesis | 5’ | | IARS-5’probe-F1 | TCCACATAAAAGCTGCCCTG | 386 |
| | | | IARS-5’probe-R1 | CAACCACCACCACTCTCTAA | |
| | 3’ | | IARS-3’probe-F1 | TACCAATCACCCACTTCCCA | 389 |
| | | | IARS-3’probe-R1 | CCAACGAGACATATCAGCAC | |
| | DT-A | | DT-A-probe-F1 | ACCGACAATAAATACGACGC | 376 |
| | | | DT-A-probe-R1 | ACTCATACATCGCATCTTGG | |

## Slide 6
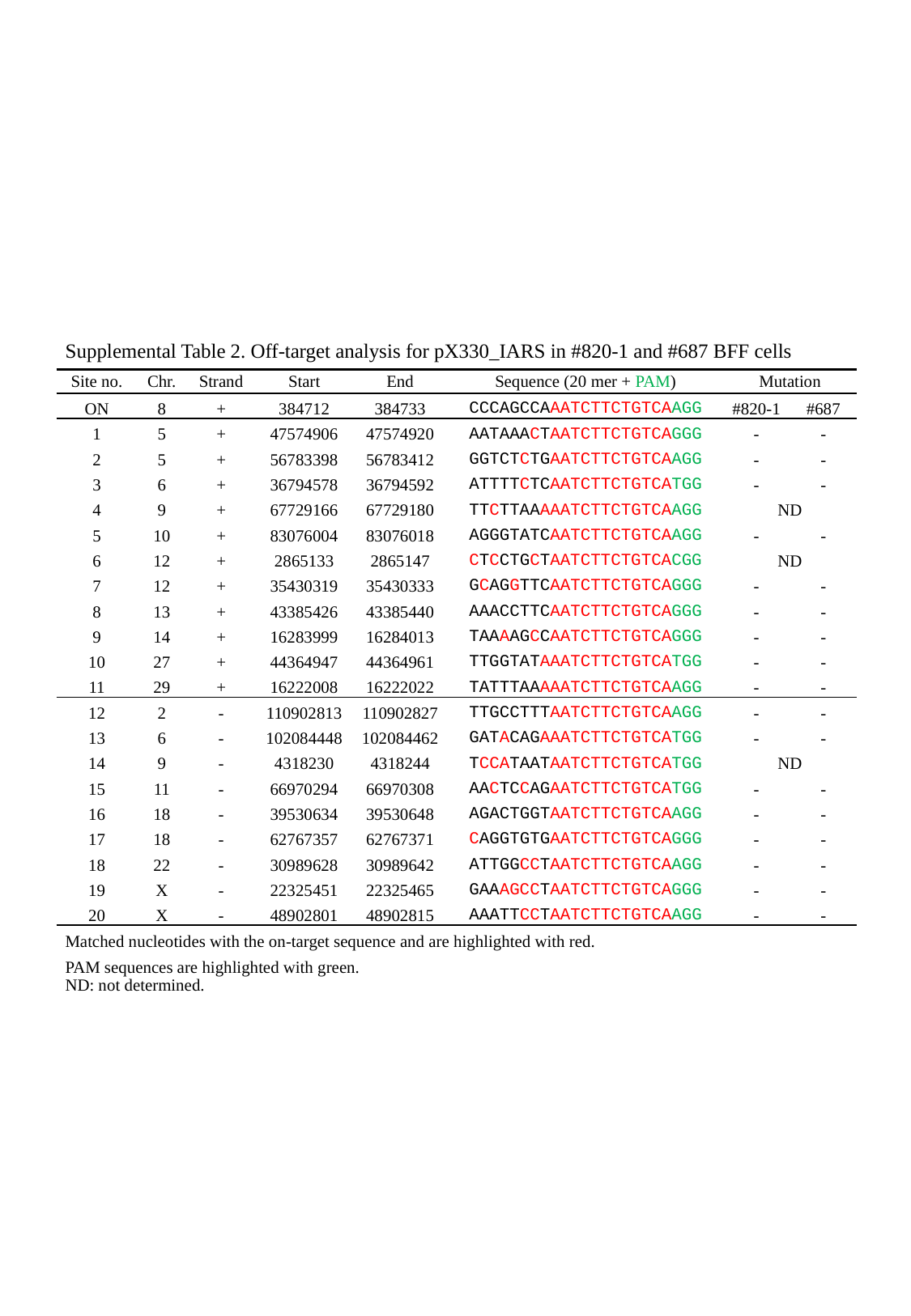

| Supplemental Table 2. Off-target analysis for pX330\_IARS in #820-1 and #687 BFF cells | | | | | | | |
| --- | --- | --- | --- | --- | --- | --- | --- |
| Site no. | Chr. | Strand | Start | End | Sequence (20 mer + PAM) | Mutation | |
| ON | 8 | + | 384712 | 384733 | CCCAGCCAAATCTTCTGTCAAGG | #820-1 | #687 |
| 1 | 5 | + | 47574906 | 47574920 | AATAAACTAATCTTCTGTCAGGG | - | - |
| 2 | 5 | + | 56783398 | 56783412 | GGTCTCTGAATCTTCTGTCAAGG | - | - |
| 3 | 6 | + | 36794578 | 36794592 | ATTTTCTCAATCTTCTGTCATGG | - | - |
| 4 | 9 | + | 67729166 | 67729180 | TTCTTAAAAATCTTCTGTCAAGG | ND | |
| 5 | 10 | + | 83076004 | 83076018 | AGGGTATCAATCTTCTGTCAAGG | - | - |
| 6 | 12 | + | 2865133 | 2865147 | CTCCTGCTAATCTTCTGTCACGG | ND | |
| 7 | 12 | + | 35430319 | 35430333 | GCAGGTTCAATCTTCTGTCAGGG | - | - |
| 8 | 13 | + | 43385426 | 43385440 | AAACCTTCAATCTTCTGTCAGGG | - | - |
| 9 | 14 | + | 16283999 | 16284013 | TAAAAGCCAATCTTCTGTCAGGG | - | - |
| 10 | 27 | + | 44364947 | 44364961 | TTGGTATAAATCTTCTGTCATGG | - | - |
| 11 | 29 | + | 16222008 | 16222022 | TATTTAAAAATCTTCTGTCAAGG | - | - |
| 12 | 2 | - | 110902813 | 110902827 | TTGCCTTTAATCTTCTGTCAAGG | - | - |
| 13 | 6 | - | 102084448 | 102084462 | GATACAGAAATCTTCTGTCATGG | - | - |
| 14 | 9 | - | 4318230 | 4318244 | TCCATAATAATCTTCTGTCATGG | ND | |
| 15 | 11 | - | 66970294 | 66970308 | AACTCCAGAATCTTCTGTCATGG | - | - |
| 16 | 18 | - | 39530634 | 39530648 | AGACTGGTAATCTTCTGTCAAGG | - | - |
| 17 | 18 | - | 62767357 | 62767371 | CAGGTGTGAATCTTCTGTCAGGG | - | - |
| 18 | 22 | - | 30989628 | 30989642 | ATTGGCCTAATCTTCTGTCAAGG | - | - |
| 19 | X | - | 22325451 | 22325465 | GAAAGCCTAATCTTCTGTCAGGG | - | - |
| 20 | X | - | 48902801 | 48902815 | AAATTCCTAATCTTCTGTCAAGG | - | - |
| Matched nucleotides with the on-target sequence and are highlighted with red. PAM sequences are highlighted with green. ND: not determined. | | | | | | | |

## Slide 7
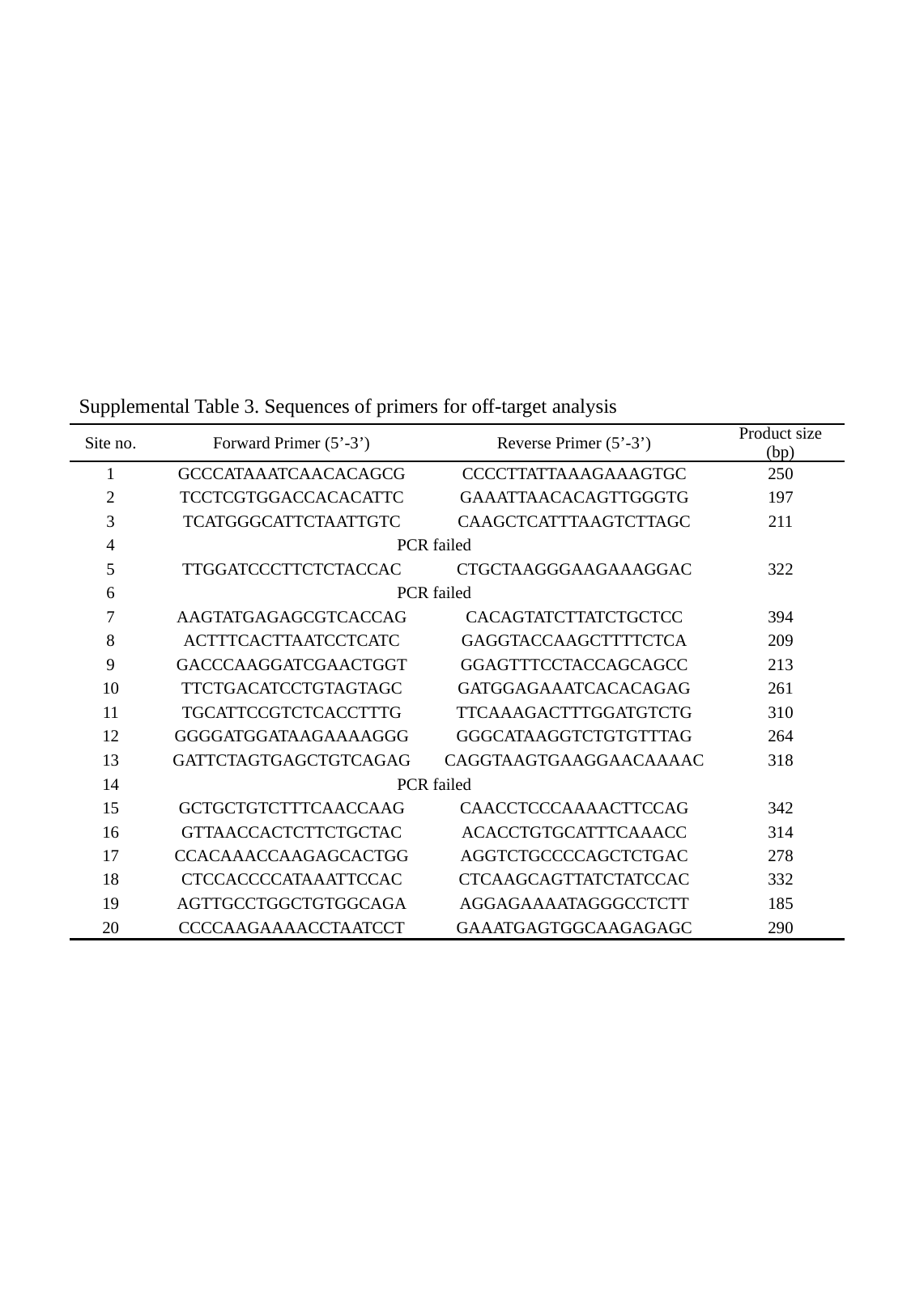

| Supplemental Table 3. Sequences of primers for off-target analysis | | | |
| --- | --- | --- | --- |
| Site no. | Forward Primer (5’-3’) | Reverse Primer (5’-3’) | Product size (bp) |
| 1 | GCCCATAAATCAACACAGCG | CCCCTTATTAAAGAAAGTGC | 250 |
| 2 | TCCTCGTGGACCACACATTC | GAAATTAACACAGTTGGGTG | 197 |
| 3 | TCATGGGCATTCTAATTGTC | CAAGCTCATTTAAGTCTTAGC | 211 |
| 4 | PCR failed | | |
| 5 | TTGGATCCCTTCTCTACCAC | CTGCTAAGGGAAGAAAGGAC | 322 |
| 6 | PCR failed | | |
| 7 | AAGTATGAGAGCGTCACCAG | CACAGTATCTTATCTGCTCC | 394 |
| 8 | ACTTTCACTTAATCCTCATC | GAGGTACCAAGCTTTTCTCA | 209 |
| 9 | GACCCAAGGATCGAACTGGT | GGAGTTTCCTACCAGCAGCC | 213 |
| 10 | TTCTGACATCCTGTAGTAGC | GATGGAGAAATCACACAGAG | 261 |
| 11 | TGCATTCCGTCTCACCTTTG | TTCAAAGACTTTGGATGTCTG | 310 |
| 12 | GGGGATGGATAAGAAAAGGG | GGGCATAAGGTCTGTGTTTAG | 264 |
| 13 | GATTCTAGTGAGCTGTCAGAG | CAGGTAAGTGAAGGAACAAAAC | 318 |
| 14 | PCR failed | | |
| 15 | GCTGCTGTCTTTCAACCAAG | CAACCTCCCAAAACTTCCAG | 342 |
| 16 | GTTAACCACTCTTCTGCTAC | ACACCTGTGCATTTCAAACC | 314 |
| 17 | CCACAAACCAAGAGCACTGG | AGGTCTGCCCCAGCTCTGAC | 278 |
| 18 | CTCCACCCCATAAATTCCAC | CTCAAGCAGTTATCTATCCAC | 332 |
| 19 | AGTTGCCTGGCTGTGGCAGA | AGGAGAAAATAGGGCCTCTT | 185 |
| 20 | CCCCAAGAAAACCTAATCCT | GAAATGAGTGGCAAGAGAGC | 290 |

## Slide 8
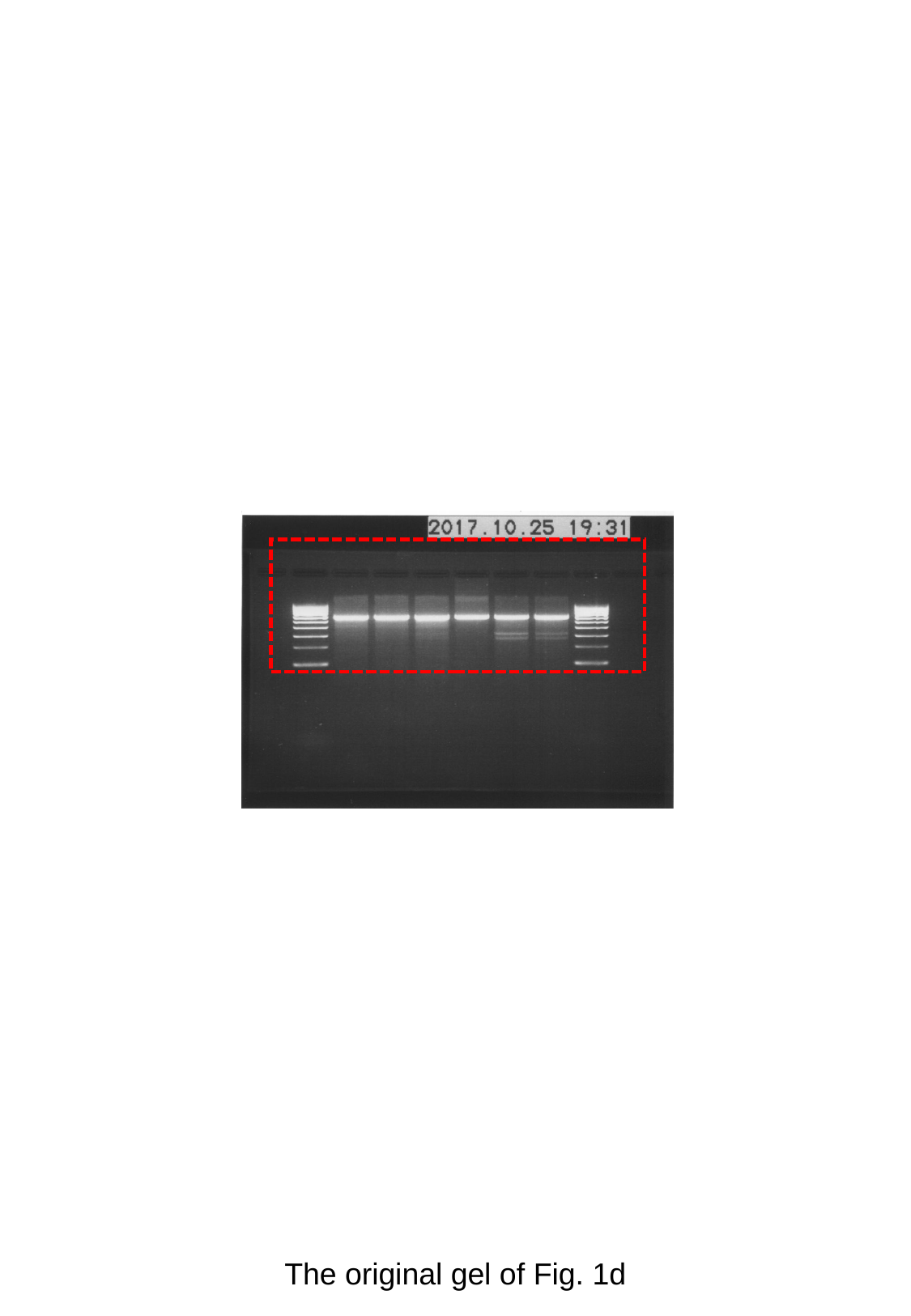

The original gel of Fig. 1d

## Slide 9
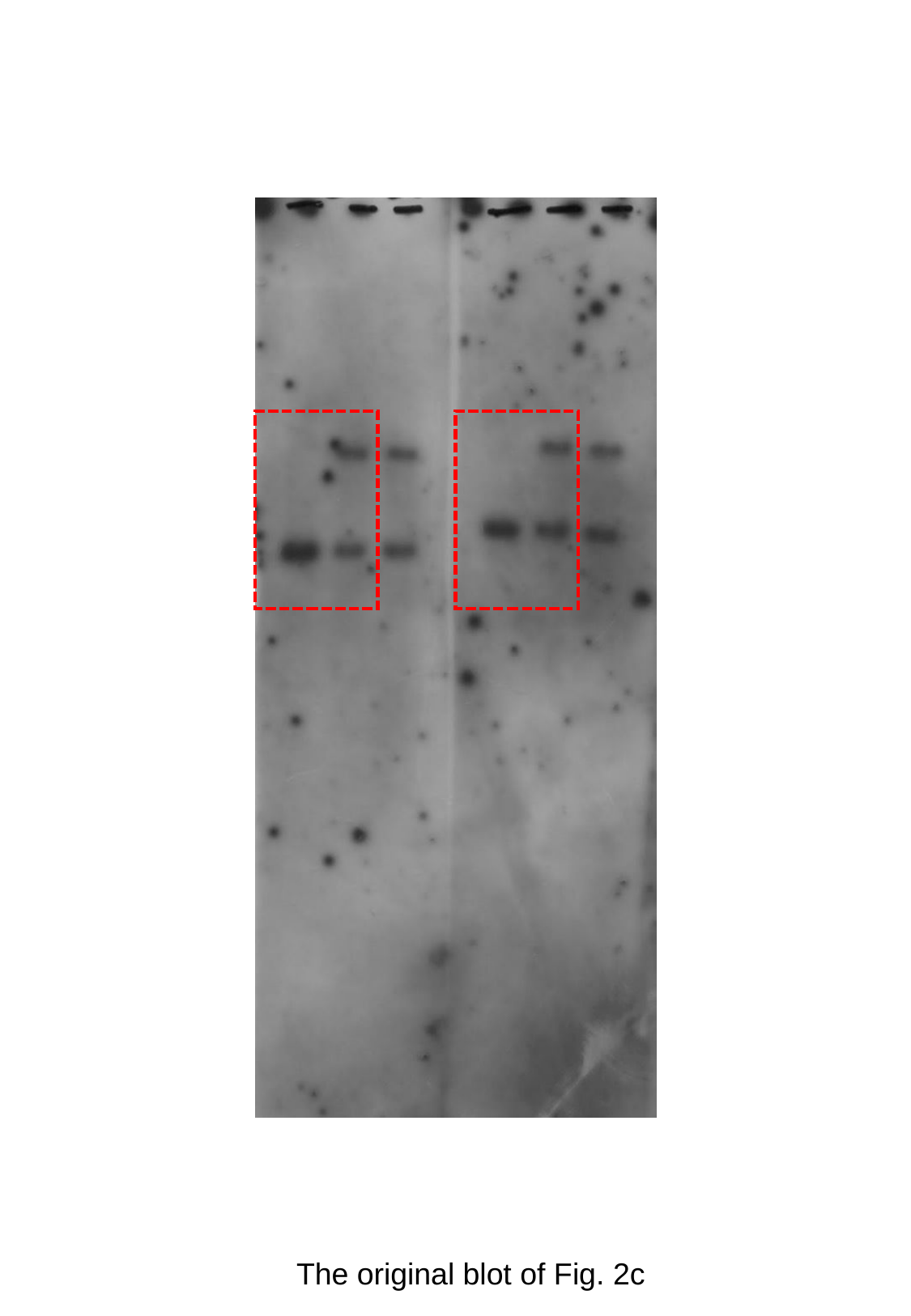

The original blot of Fig. 2c

## Slide 10
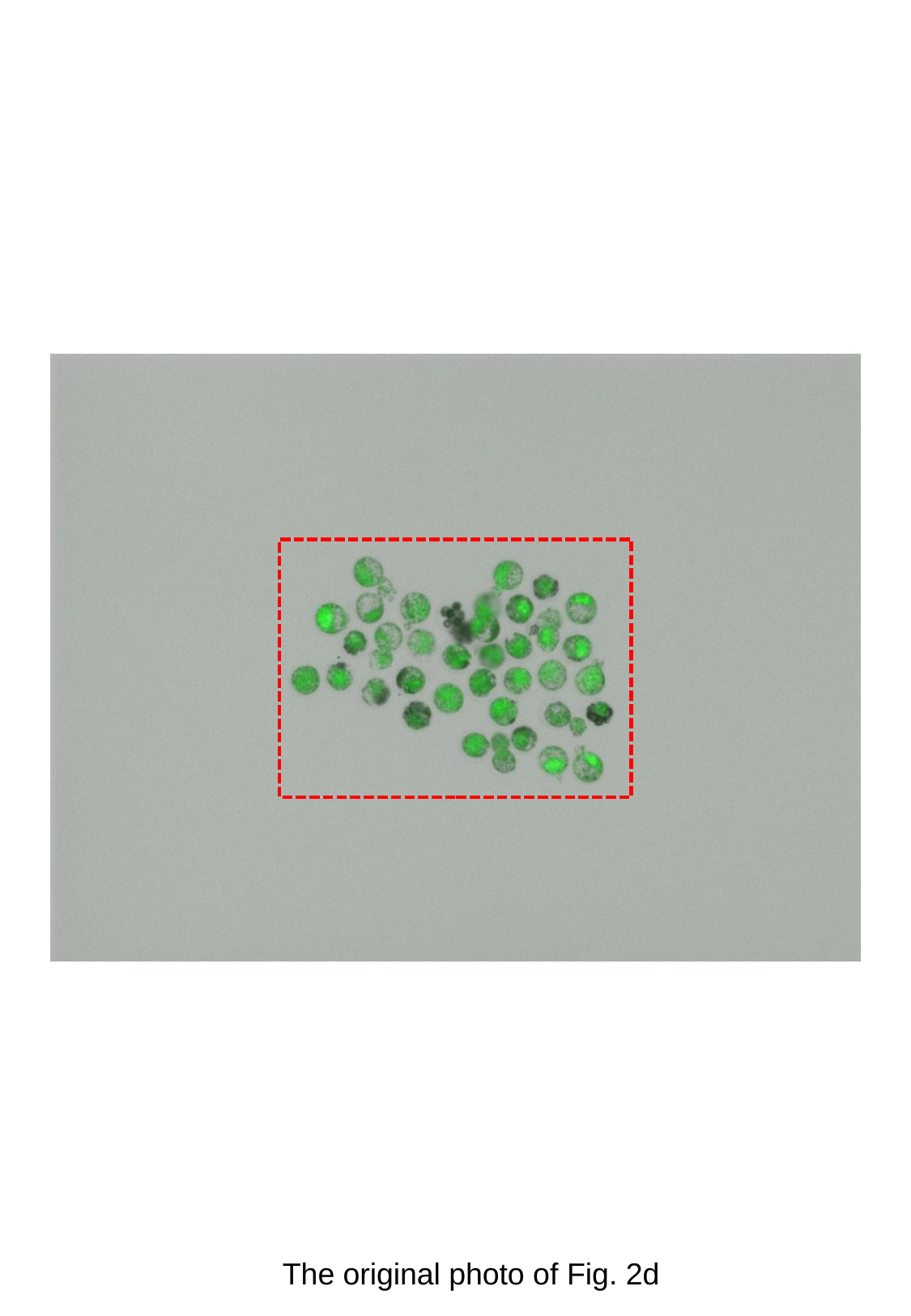

The original photo of Fig. 2d

## Slide 11
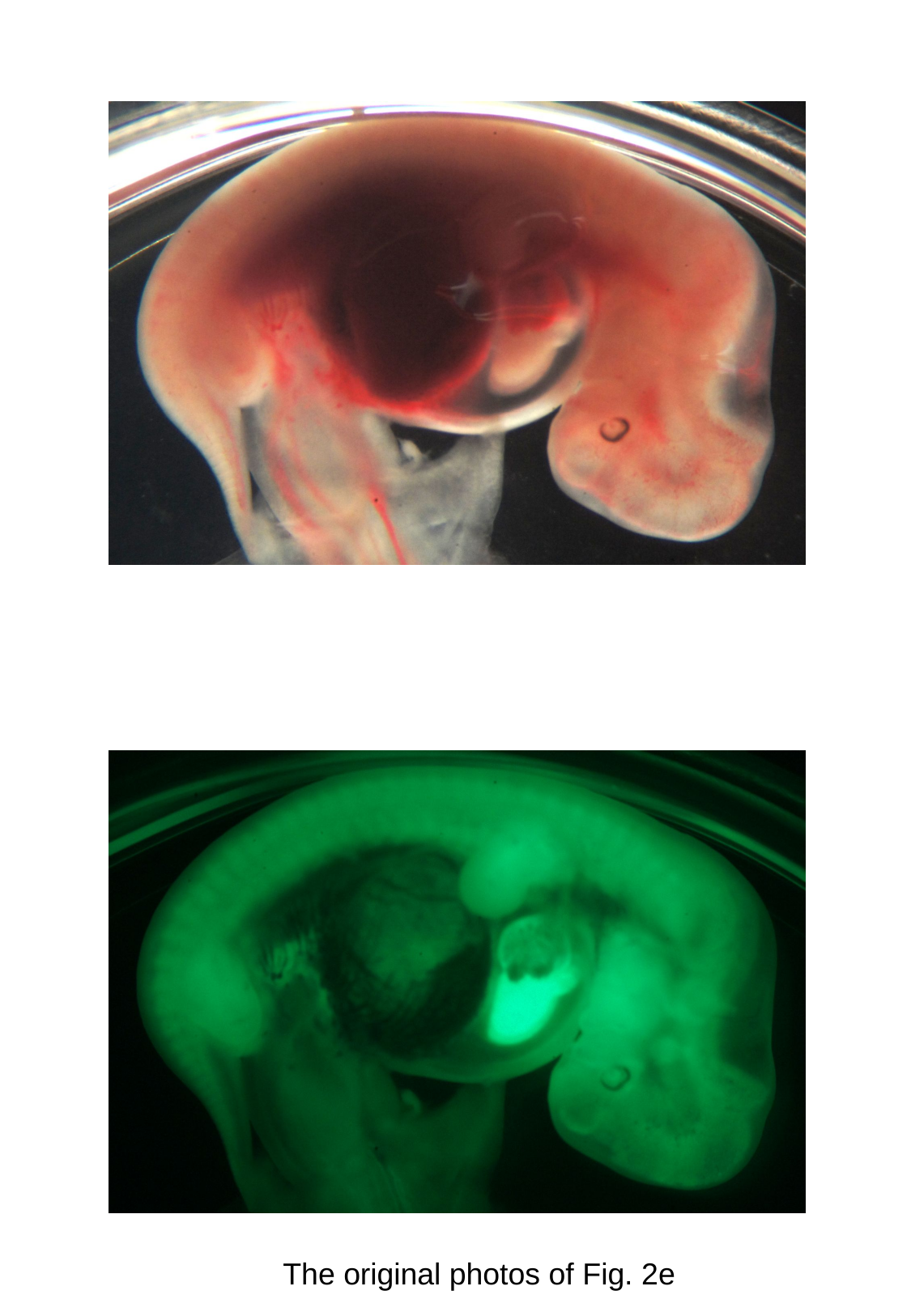

The original photos of Fig. 2e

## Slide 12
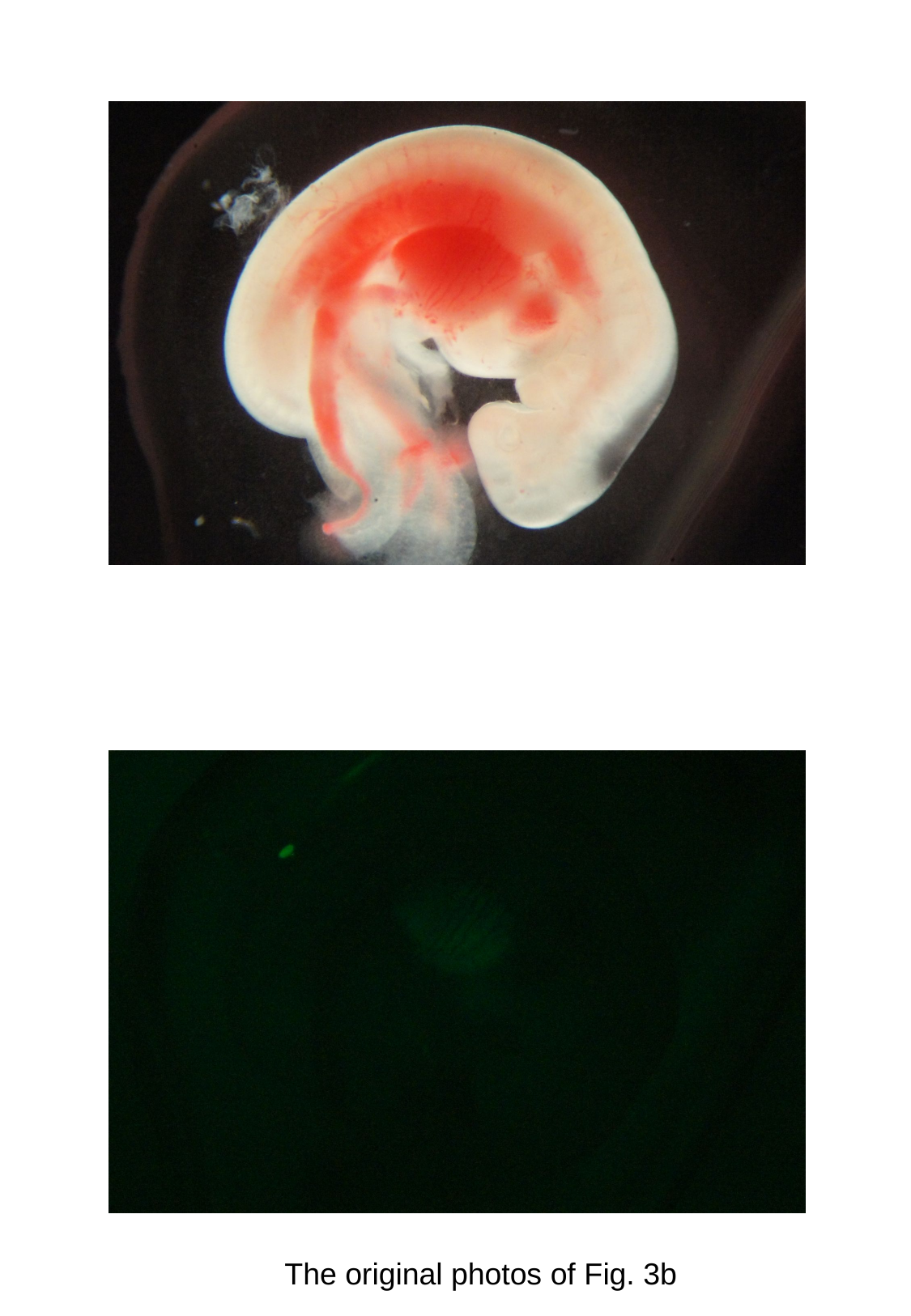

The original photos of Fig. 3b

## Slide 13
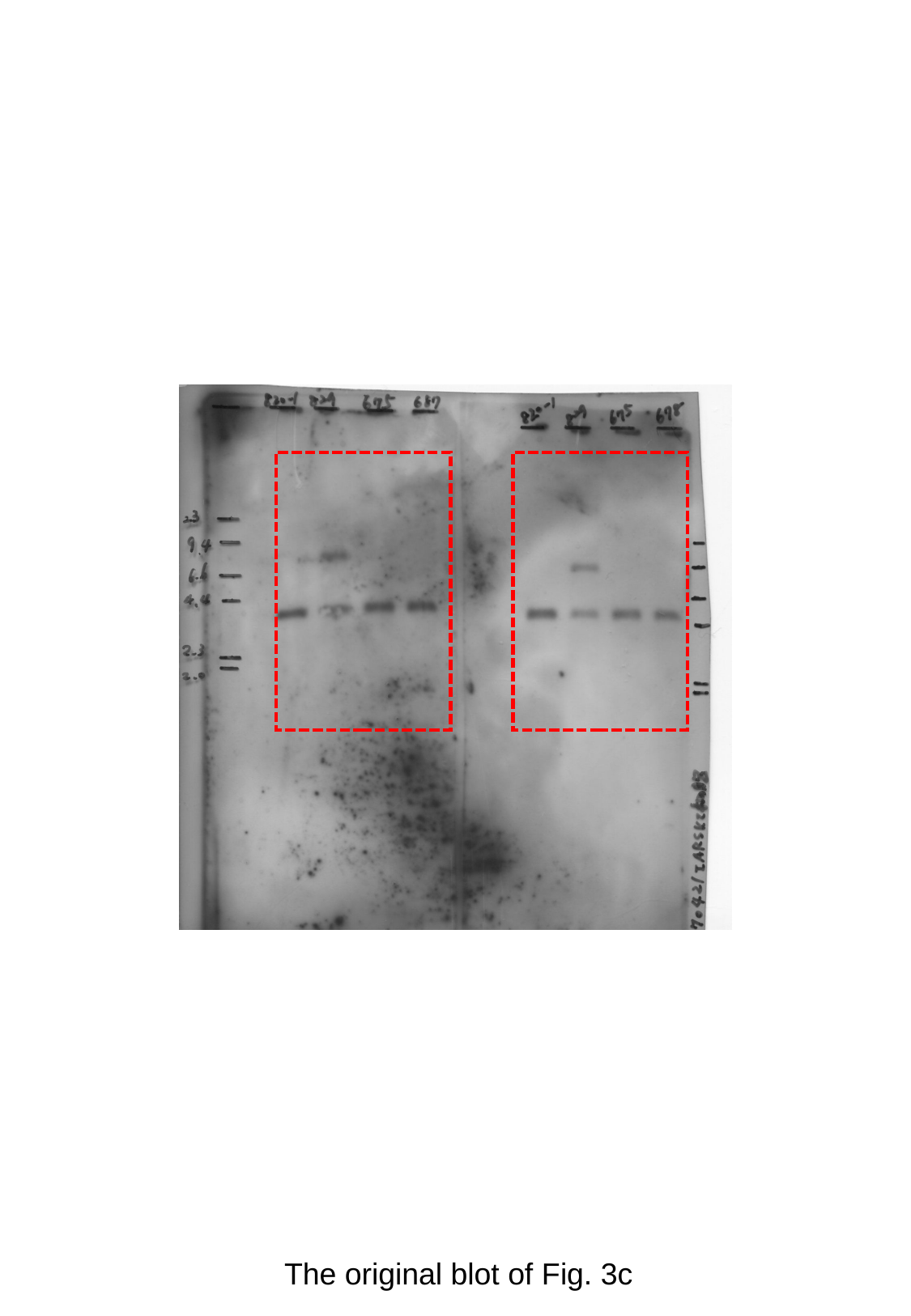

The original blot of Fig. 3c

## Slide 14
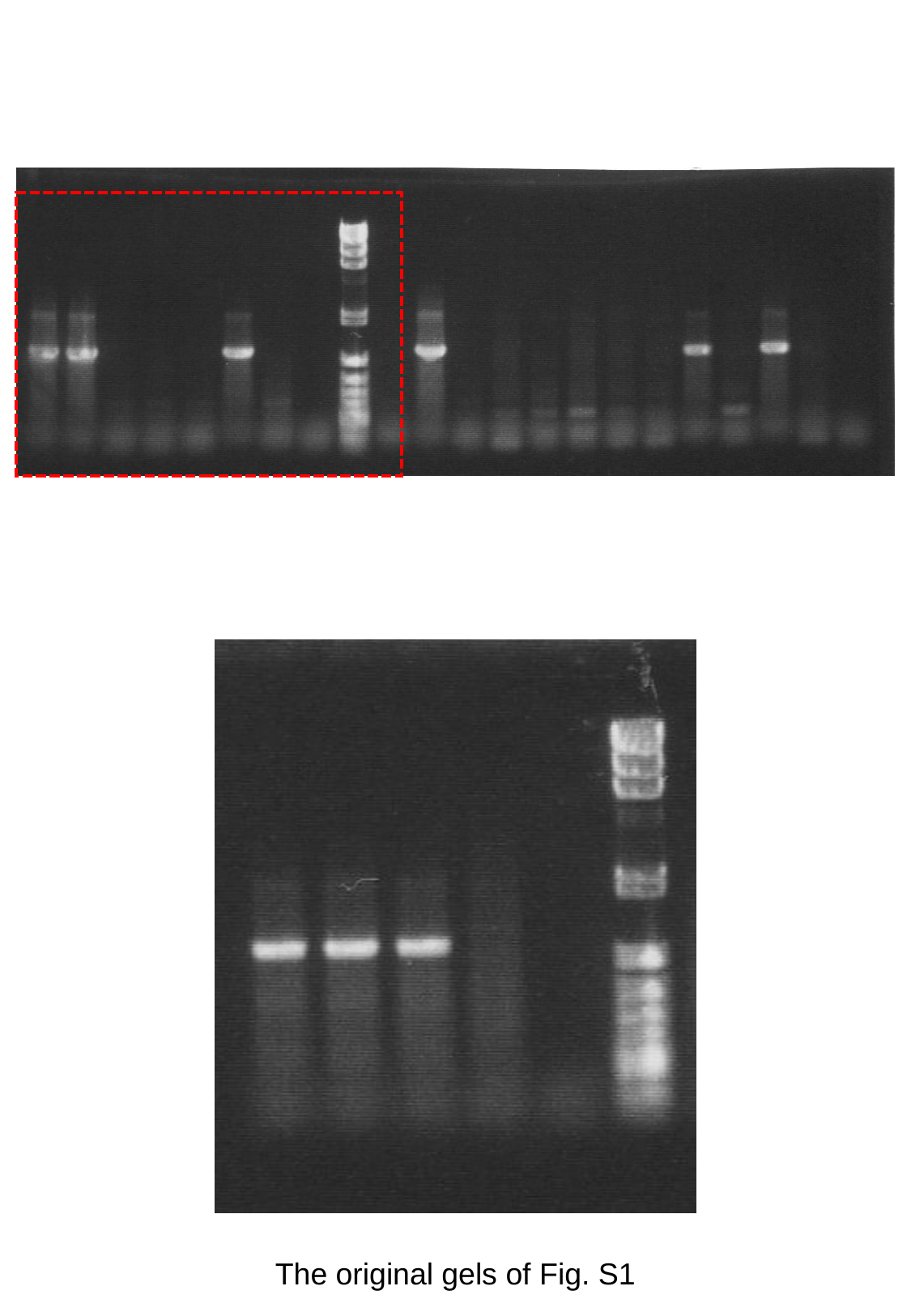

The original gels of Fig. S1

## Slide 15
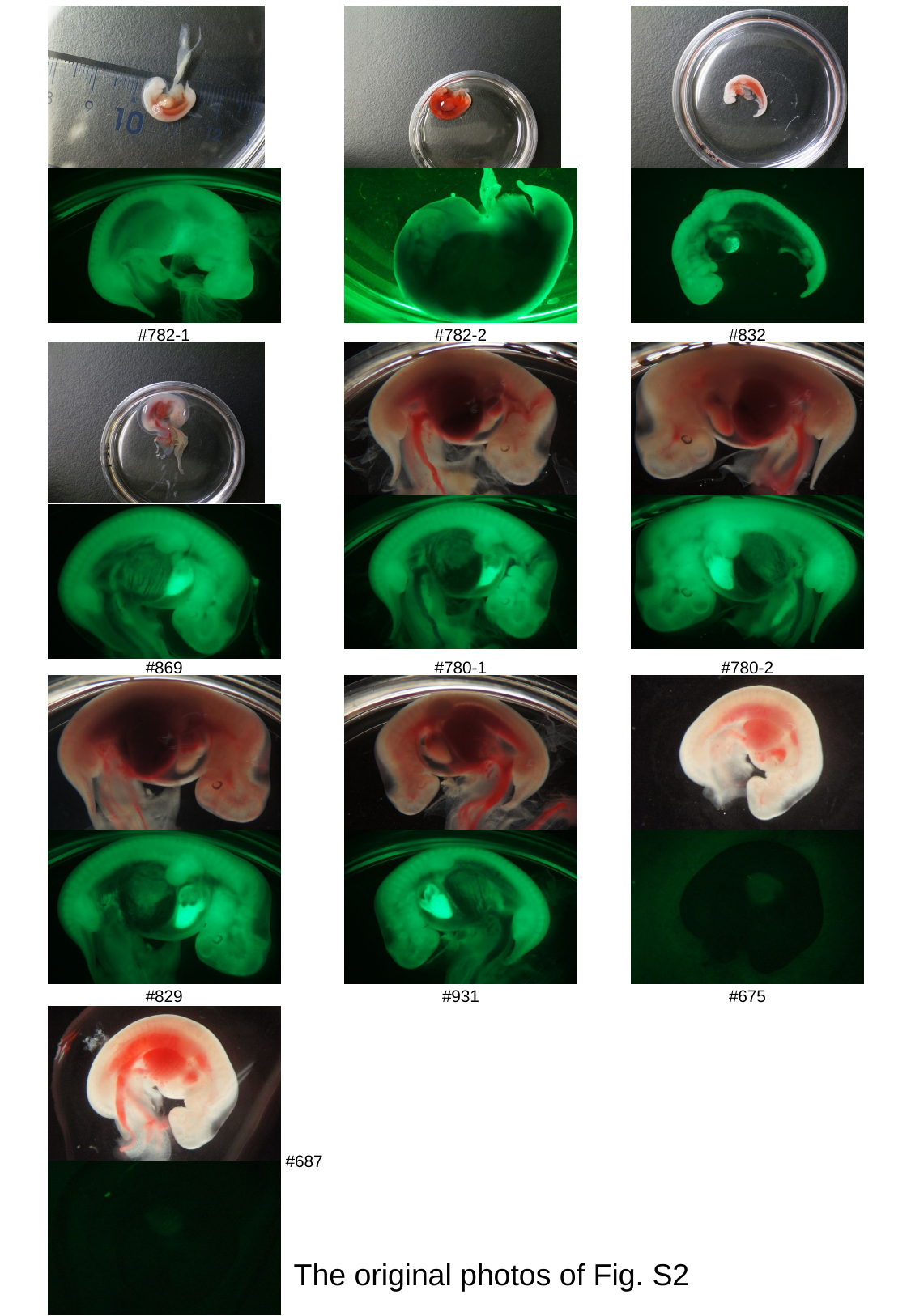

#782-1
#782-2
#832
#869
#780-1
#780-2
#829
#931
#675
#687
The original photos of Fig. S2

## Slide 16
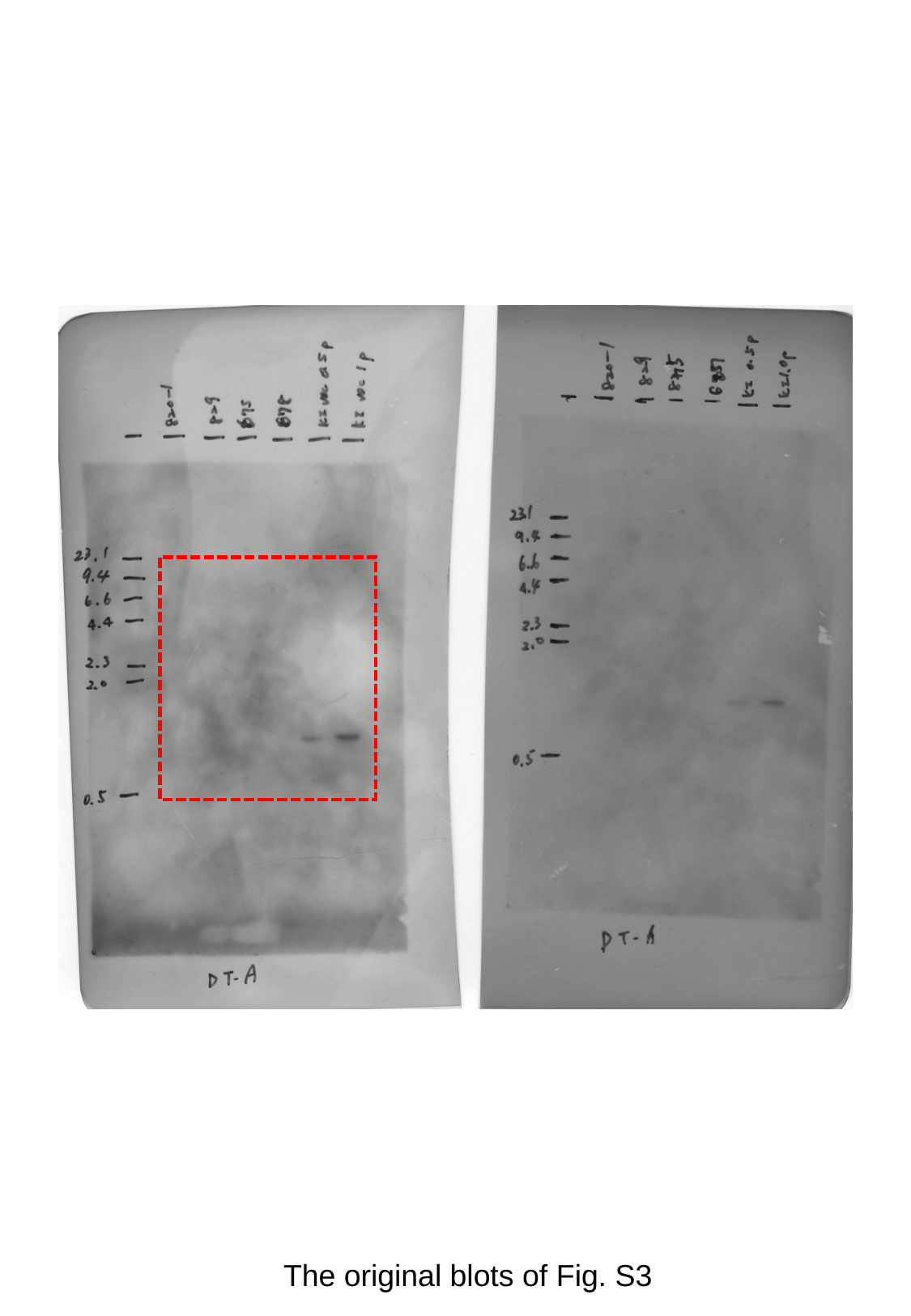

The original blots of Fig. S3
